# PD-1 deficiency exacerbates *Mycobacteroides abscessus* lung infection via metabolic rewiring and dysregulated neutrophil/T cell responses

**DOI:** 10.3389/fimmu.2026.1803806

**Published:** 2026-04-24

**Authors:** Xiaowen Yu, Lu Huang, Jianping Xie

**Affiliations:** 1 Institute of Modern Biopharmaceuticals, School of Life Sciences, Southwest University, Chongqing, China; 2 Basic Research Laboratory of Traditional Chinese Medicine, Chongqing Traditional Chinese Medicine Hospital, Chongqing University of Chinese Medicine., Chongqing, China; 3 Department of Pathology, Xinqiao Hospital, Army Medical University, Chongqing, China

**Keywords:** CD4^+^ T cell, metabolic reprogramming, *Mycobacteroides abscessus*, neutrophil, PD-1

## Abstract

**Background:**

Pulmonary *Mycobacteroides abscessus* (MAB) infection presents a therapeutic challenge, and while anti-programmed cell death protein 1(PD-1) therapy is clinically associated with increased MAB risk, yet the underlying immunomodulatory mechanisms remain elusive.

**Methods:**

We established a PD-1-deficient mouse model of MAB infection and assessed bacterial clearance, immune responses, and metabolic alterations using colony counting, histopathology, flow cytometry, multiplex immunofluorescence, transcriptomics, and metabolic assays.

**Results:**

PD-1^−/−^ mice showed increased susceptibility to MAB. Early infection was marked by elevated lung CD4^+^ T cells (Th17/Treg), diminished Th1 cells and neutrophils, and increased blood cytokines (IFN-γ, IL-21, IL-6, IL-27). Late infection featured further Th1 reduction, neutrophil accumulation, and NETs activation (H3cit, NE-DNA, MPO), along with reduced CXCL9 but elevated IL-6, IL-21, IL-27, CXCL10, and S100A8 in blood. Metabolic changes included decreased blood glucose, elevated lung ATP, and upregulation of HIF-1 pathway proteins (e.g., HK2, iNOS). *In vitro*, PD-1 deficiency suppressed glycolysis in T cells yet augmented it in neutrophils upon MAB infection.

**Conclusion:**

PD-1 deficiency disrupts T cell–neutrophil crosstalk via metabolic reprogramming, resulting in immune dysregulation and exacerbated infection. These findings underscore infection risks linked to PD-1/PD-L1 blockade, informing clinical safety and therapeutic strategies.

## 1 Introduction

MAB, a rapidly growing non-tuberculous mycobacterium (NTM), causes pulmonary, cutaneous, and soft tissue infections, particularly in immunocompromised individuals, those with underlying lung disease, or following surgery or trauma (1, 2). However, MAB exploits multiple virulence mechanisms to acquire nutrients and evade host immunity, including modulating surface lipids composition, upregulating efflux pumps, and deploying secretion systems (3). It is intrinsically resistant to many antibiotics, including macrolides, aminoglycosides, rifamycins, tetracyclines, and β-lactams (1). With treatment success rates below 30% (4), MAB infections are often deemed an ‘antibiotic nightmare’ (5). Host immune responses play a decisive role in controlling MAB infection, yet the underlying mechanisms remain incompletely elucidated, particularly regarding immune checkpoints.

PD-1, a B7/CD28 superfamily inhibitory co-stimulatory molecule encoded by *Pdcd1*, is broadly expressed on T cells, B cells, and macrophages. Binding of PD-1 to its ligand PD-L1 triggers downstream signaling that suppresses T cell proliferation, activation, and cytokine secretion, modulates cellular metabolism and cytotoxic activity, and can promote apoptosis of activated T cells (6). Monoclonal antibodies targeting PD-1 or PD-L1 counteract this immunosuppression, restoring antitumor T cell function and enabling tumor control. Such agents have shown clinical efficacy in malignancies such as melanoma, non-small cell lung cancer, and urothelial carcinoma (7). Beyond oncology, PD-1 blockade has shown therapeutic promise in infectious diseases, particularly in Epstein–Barr virus (EBV)-associated hemophagocytic lymphohistiocytosis (8) and chronic active EBV infection after stem cell transplantation (9). However, PD-1/PD-L1 inhibition also poses risks: retrospective studies have reported treatment-related adverse events in patients with *Mycobacterium tuberculosis* (MTB) and NTM infections (10). A meta-analysis further confirmed that such inhibition significantly increases the risk of MTB reactivation and tuberculosis-related mortality (11). The immunometabolic mechanisms underlying these divergent roles of PD-1 blockade across infectious contexts remain poorly understood.

In 2021, Okamoto et al. reported a lung cancer patient with partial tumor regression from PD-1 inhibitor plus chemotherapy, yet pulmonary disease progressed due to subsequent MAB infection (12). Recent studies show that lung cancer patients on immune checkpoint inhibitors (ICIs) experience earlier pulmonary NTM infection, primarily from the MAB complex (13). In contrast, another patient with lung cancer and MAB subsp. marseillensis lung disease exhibited nodular improvement and positive tumor response after two months of anti-PD-1 nivolumab therapy (14). These contrasting outcomes drive further study of PD-1’s immunomodulatory role in host defense against MAB infection.

Accordingly, We hypothesize that PD-1 signaling plays a protective role in host defense against MAB infection by modulating immune cell activity and metabolic homeostasis. To test this hypothesis, we established a mouse model of MAB infection and assessed how PD-1 deficiency affects pulmonary bacterial clearance, immune cell recruitment and function, and metabolic reprogramming. Our findings reveal that PD-1 deletion dynamically modulates the infiltration and effector responses of CD4^+^ T cells and neutrophils in the lungs during infection, which is closely linked to immune cell metabolic reprogramming.

## 2 Materials and methods

### 2.1 Strains

The *M. abscessus* ATCC 19977 strain was generously provided by Dr Wang from Daping Hospital, Army Medical University. Bacteria were cultured at 37°C in 7H9 broth or on 7H9 agar, both supplemented with 0.5% (v/v) glycerol and 0.05% (v/v) Tween 80. For long-term storage, the strain was preserved at –80°C in 10% sterile glycerol. For experimental use, *M. abscessus* ATCC 19977 was grown to mid-log phase (OD_600_ = 0.8) in 7H9 broth, washed and resuspended in PBS or Clone E6–1 Cell Complete Medium, and adjusted to an OD_600_ of 0.5 or 0.3 to prepare the bacterial suspension.

### 2.2 Mice

PD-1^−/−^ mice were kindly provided by Professor Long (Xinqiao Hospital, Army Medical University). Wild-type (WT) C57BL/6 mice (6–8 weeks old) were purchased from Vital River (China). All mice were maintained under specific pathogen-free (SPF) conditions in the animal facility of Southwest University. They were housed in a temperature-controlled (22 ± 1°C) and humidity-controlled (60 ± 5%) environment under a 12-hour light/dark cycle. Mice were group-housed (up to 5 per cage) in individually ventilated cages (IVCs) supplied with standard bedding, nesting material, and PVC tunnels. Food and water were available ad libitum. After MAB infection, animals were monitored daily for body weight, food and water consumption, and signs of pain or distress. Mice showing severe and unrelieved distress or those at the experimental endpoint were euthanized via intraperitoneal injection of Zoletil 50 (50 mg/kg) and cervical dislocation.

### 2.3 Experimental mice model of MAB infection

Eight-week-old mice, with equal numbers of males and females, were intravenously administered 2×10^7^ CFU of mid-logarithmic phase MAB and monitored daily for survival.

### 2.4 Jurkat cell line, Jurkat cell activation and MAB infection

The human acute T lymphoblastic leukemia Jurkat T cell line was provided by Professor Wang (Chongqing Traditional Chinese Medicine Hospital). Cells were maintained in Clone E6–1 complete medium (Procell system, China) at 37°C with 5% CO_2_.

For activation, Jurkat cells were seeded in 12-well plates at 5×10^5^ cells/well and stimulated with Human CD3/CD28 T Cell Activation Beads (BioLegend, USA) for 24 h. After bead removal, cells were infected with MAB at an MOI of 10 for 4 h in antibiotic-free medium. Following infection, cells were washed with PBS containing penicillin/streptomycin, replenished with complete medium, and further incubated at 37°C with 5% CO_2_ for 24, 48, and 72 h.

### 2.5 Neutrophil isolation

Murine bone marrow was flushed out of the tibia and the femur in 0.9% NaCl (Kelun, Chengdu, China) using a 23G needle (WEGO, China) and passed through a 70 um cell strainer (Biosharp, China) Bone marrow neutrophils were obtained with mouse marrow neutrophil isolation kit (Solarbio|, China), following the manufacturer’s instructions.

### 2.6 Neutrophils MAB infection

Neutrophils were seeded at 1×10^6^ cells/well and directly infected with MAB at MOI 10 for 30 min in antibiotic-free medium. After infection, all cells were washed with PBS (Biosharp, China) containing penicillin/streptomycin(Biosharp, China), replenished with complete medium, and incubated at 37°C with 5% CO₂ for 0.5 h, 2.5 h, 6 h.

### 2.7 Gene silencing by siRNA

To knock down the expression of *Pdcd1*, Jurkat cells were transfected with siRNA (GenePharma, Cat# 25308664) using siRNA mate plus Transfection Kit (GenePharma, China) following the manufacturer’s instructions. A non-targeting control siRNA (GenePharma, Cat# 250720) was used in parallel. The sequence of siRNA that target *Pdcd1* and non-targeting control siRNA listed in **Table 1**. The final siRNA concentration was 30 pmol. Cells were harvested 24 h post-transfection for subsequent analyses. The knockdown efficiency, exceeding 80%, was verified by qRT-PCR (see SF7).

**Table 1:** The sequence of siRNA enrolled in the study.

Name	Sense (5’-3’)	Antisense (5’-3’)
PDCD1-Homo-115	CCAGGAUGGUUCUUAGACUTT	AGUCUAAGAACCAUCCUGGTT
PDCD1-Homo-247	CUAAACUGGUACCGCAUGATT	UCAUGCGGUACCAGUUUAGTT
PDCD1-Homo-793	GAGUAUGCCACCAUUGUCUTT	AGACAAUGGUGGCAUACUCTT
NC	UUCUCCGAACGUGUCACGUTT	ACGUGACACGUUCGGAGAATT

### 2.8 ELISA

Serum levels of IFN-γ, IL-2, IL-6, IL-27, NE-DNA, MPO-DNA, MPO, CXCL9, CXCL2, CXCL10, and S100A8 were quantified using commercial immunoassays (Meimmian, China) according to the manufacturer’s protocol.

### 2.9 Flow cytometry analysis

Lungs were perfused with RPMI-1640 and digested at 37 °C for 30 min with 0.25 mg/mL Liberase™ (Sigma, USA) and 0.25 mg/mL DNase I(Solarbio, China).

All antibodies for flow cytometry were commercially acquired from BioLegend. Single-cell suspensions were stained in FACS buffer using antibodies against the following mouse proteins: CD45 (clone 30-F11), Ly6G (clone 1A8), CD11c (clone N418), CD11b (clone M1/70), Ly6C (clone HK1.4), CD4 (clone GK1.5), CD8a (clone 53-6.7), CD3 (clone 17A2), I-A/I-E (clone M5/114.15.2), CD64 (clone S18017D), MerTk (clone 2B10C42), CD19 (clone 6D5), NK-1.1 (clone PK136), CXCR5 (clone L138D7), ICOS (clone C398.4A), CD69 (clone H1.2F3), and CD62L (clone MEL-14). Staining was performed for 30 minutes at room temperature. After incubation, cells were washed and resuspended in FACS buffer.

For intracellular cytokine staining, cells were fixed and permeabilized, followed by incubation with antibodies against CD45, CD3, CD4 (clone GK1.5), IFN-γ (clone W18272D), IL-4 (clone 11B11), and IL-17A (clone TC11-18H10.1). Prior to staining, cells were stimulated for 4 hours with PMA and brefeldin A (MULTI SCIENCE, China).

For Treg cell detection, cells were first stained surface markers CD45, CD4, and CD25 (clone PC61) for 30 minutes at room temperature. After fixation and permeabilization using a Perm/Fix solution (BioLegend, USA), intracellular staining for Foxp3 (clone MF-14) was performed according to the manufacturer’s protocol. Stained cells were analyzed on a Beckman Coulter flow cytometer.

### 2.10 Multiplex immunofluorescence analyses

Mouse lung tissues were sectioned into 4-mm-thick slices. Immunostaining was conducted on the slides using antibodies against Ly6G (1:3000, GB11229, Servicebio, China), MPO (1:5000, GB150006, Servicebio, China), and H3cit (1:3000, AB281584, Abcam, USA). Primary antibodies were incubated at 37°C for 1 h. Secondary antibodies (Servicebio, China) were applied at 1:300 dilution as needed. DNA was stained with Hoechst 33258 (G1011, Servicebio, China). Slides were scanned using a Pannoramic MIDI system (3DHISTECH, Hungary) and analyzed with ImageJ software.

### 2.11 Histopathological analysis and scoring

Mice were infected and euthanized at designated time points. Liver, lung, and spleen tissues were collected, fixed in 4% PFA, stained with hematoxylin and eosin, and examined using a Leica microscope. Histological lesions were scored as: 0, normal; 1, mild, identifiable but minimal; 2, moderate, clearly evident and progressive; 3, severe, extensive involvement of most tissue.

### 2.12 RT-PCR

RNA was isolated from the lungs of WT and PD-1^−/−^ mice using a RNA extraction kit(Servicebio, China). PCR was performed on a Bio-Rad IQ5 system with the following cycling conditions: 95°C for 5 min, then 40 cycles of 95°C for 30 s, 58°C for 30 s, and 72°C for 30 s.

All qPCR assays were performed in triplicate. Cycle threshold (CT) values of target genes were normalized to GAPDH, yielding ΔCT values. Relative gene expression was determined by the 2^−ΔΔCT^ method. Primer sequences are listed in **Table 2**.

**Table 2:** Primers used in the study.

Primer	Sequence of Primers (5’-3’)	Gene ID
Cd4-F	GTTCAGGACAGCGACTTCTGGA	12504
Cd4-R	GAAGGAGAACTCCGCTGACTCT	
Cd3d-F	TGACCTCATCGCAACTCTGCTC	12500
Cd3d-R	TCAGCAGTGCTTGAACCTCAGC	
Cd5-F	AGAACCAGGTCTTCTGCCAAGG	12507
Cd5-R	TTGTGGGTGGAGGTGTCGTTCT	
Ifng-F	CAGCAACAGCAAGGCGAAAAAGG	15978
Ifng-R	TTTCCGCTTCCTGAGGCTGGAT	
Icos-F	GCAGCTTTCGTTGTGGTACTCC	54167
Icos-R	TGTGTTGACTGCCGCCATGAAC	
S100a9-F	TGGTGGAAGCACAGTTGGCAAC	20202
S100a9-R	CAGCATCATACACTCCTCAAAGC	
S100a8-F	CAAGGAAATCACCATGCCCTCTA	20201
S100a8-R	ACCATCGCAAGGAACTCCTCGA	
Syk-F	GAGAGCACTGTGTCCTTCAACC	20963
Syk-R	CAGCATAAGGGCTCTCGTACAC	
Itgb2i-F	TCTTCGTGGTGCCCTCAAGGAT	16415
Itgb2i-R	CTGTAGGCGTTCCTGATGAGCT	
Prtn3-F	ATCCACCCGAGATTCGTGCTGA	19152
Prtn3-R	CTGGAAGACCTGACTGATGGTG	
Cd177-F	GCAATGACCTGTCTACCACAGC	68891
Cd177-R	CGGTGCATTCTCACAGGCTTGT	
Pram1-F	GCCTTTTGGCTGGTTCCTGTGT	14289
Pram1-R	CAAATGCAGCGGTCCAAGGCAA	
Fpr2-F	GCCTTTTGGCTGGTTCCTGTGT	14289
Fpr2-R	CAAATGCAGCGGTCCAAGGCAA	
Selp-F	AAGATGCCTGGCTACTGGACAC	20344
Selp-R	CAAGAGGCTGAACGCAGGTCAT	
Itgb2-F	CTTTCCGAGAGCAACATCCAGC	16414
Itgb2-R	GTTGCTGGAGTCGTCAGACAGT	
Itgam-F	TACTTCGGGCAGTCTCTGAGTG	16409
Itgam-R	ATGGTTGCCTCCAGTCTCAGCA	
Ctsg-F	AGTCCAGAAGGGCTGAGTGCTT	13035
Ctsg-R	GCACTGTGATGAGTTGCTGGGT	
Actinb-F	CATTGCTGACAGGATGCAGAAGG	11461
Actinb-R	TGCTGGAAGGTGGACAGTGAGG	
H-Pdcd1-F	AAGGCGCAGATCAAAGAGAGCC	5133
H-Pdcd1-R	CAACCACCAGGGTTTGGAACTG	
M-Pdcd1-F	CGGTTTCAAGGCATGGTCATTGG	18566
M-Pdcd1-R	TCAGAGTGTCGTCCTTGCTTCC	

### 2.13 RNA isolation, sequencing and data analysis

Lungs were harvested from MAB-infected WT and PD−1^−/−^ mice at specified time points for RNA extraction. RNA sequencing was performed by Zhongke New Life (China) and Novogene (China). Raw image files from high−throughput sequencing were base−called using CASAVA to generate sequencing reads. These reads were aligned to the NCBI reference genome of WT C57BL/6 mice. Gene expression levels were quantified based on the number of reads mapped to each gene. To ensure comparability across genes and samples, raw read counts were normalized to generate normalized expression values.

Gene expression data (accession number: GSE317955, GSE317833) has been submitted to GEO.

### 2.14 Measurement of ATP, lactate and glucose levels

ATP content in lung tissue from mice, Jurkat cell and neutrophil was quantified using an ATP Assay Kit (Elabscience, China), following the manufacturer’s instructions, with absorbance measured on a multifunctional microplate reader (Gene, China). Similarly, lactate and glucose concentrations in lung and peripheral blood of mice, neutrophil and Jurkat cell culture supernatant were assessed using the corresponding Lactate and Glucose Assay Kits (Elabscience, China), according to the provided protocols, with readings taken on the same microplate reader.

### 2.15 Western blot

Western blotting was performed according to standard protocols. Total protein was extracted from lung using RIPA buffer(Beyotime, China) supplemented with phosphatase and protease inhibitors (Beyotime, China). Protein concentration was determined with a BCA assay kit (Epizyme, China). Equal amounts of protein were separated by SDS-PAGE and transferred onto PVDF membranes (Merck Millipore, Germany). The membranes were blocked with 5% skim milk for 1 hour and then incubated overnight at 4 °C with the following primary antibodies: HK2 (1:3000, 22029-1-AP), Adpgk (1:1000, 15639-1-AP), Il-6ra (1:5000, 23457-1-AP), iNOS (1:1000, 22226-1-AP), Angpt2 (1:5000, 83816-1-RR), β-actin(1:5000, 20536-1-AP) and Hmox1 (1:5000, 10701-1-AP). Antibodies used in Western blot analysis were purchased from Proteintech(China). After three washes with TBST, membranes were incubated with corresponding secondary antibodies(1:2000, SA00001-2) for 1 hour at room temperature. Protein bands were visualized using an Electrochemiluminescence Plus Reagent Kit (Epizyme, China) and analyzed by densitometry with Image Lab software.

### 2.16 Ethical issues

Mice were housed and treated at the Southwest University animal facility. All procedures followed institutional guidelines and were approved by the Institutional Animal Care and Use Committee of Southwest University(approval No. IACUC-20230512-07).

### 2.17 Statistical analysis

All experiments were performed in triplicate. Statistical analyses were conducted using Prism (v8.0.2; GraphPad Software). After confirming normality, comparisons between two conditions were made with unpaired two-tailed Student’s t-tests. Multiple comparisons were performed using one-way ANOVA with Tukey’s correction. Survival curves were compared by the log-rank test. Data are expressed as mean ± SD from at least three independent biological replicates. Significance levels are denoted as ***p < 0.001, **p < 0.01, *p < 0.05; ns indicates not significant.

## 3 Results

### 3.1 PD-1^−/−^ mice were highly susceptible to MAB infection

**Figure 1:**
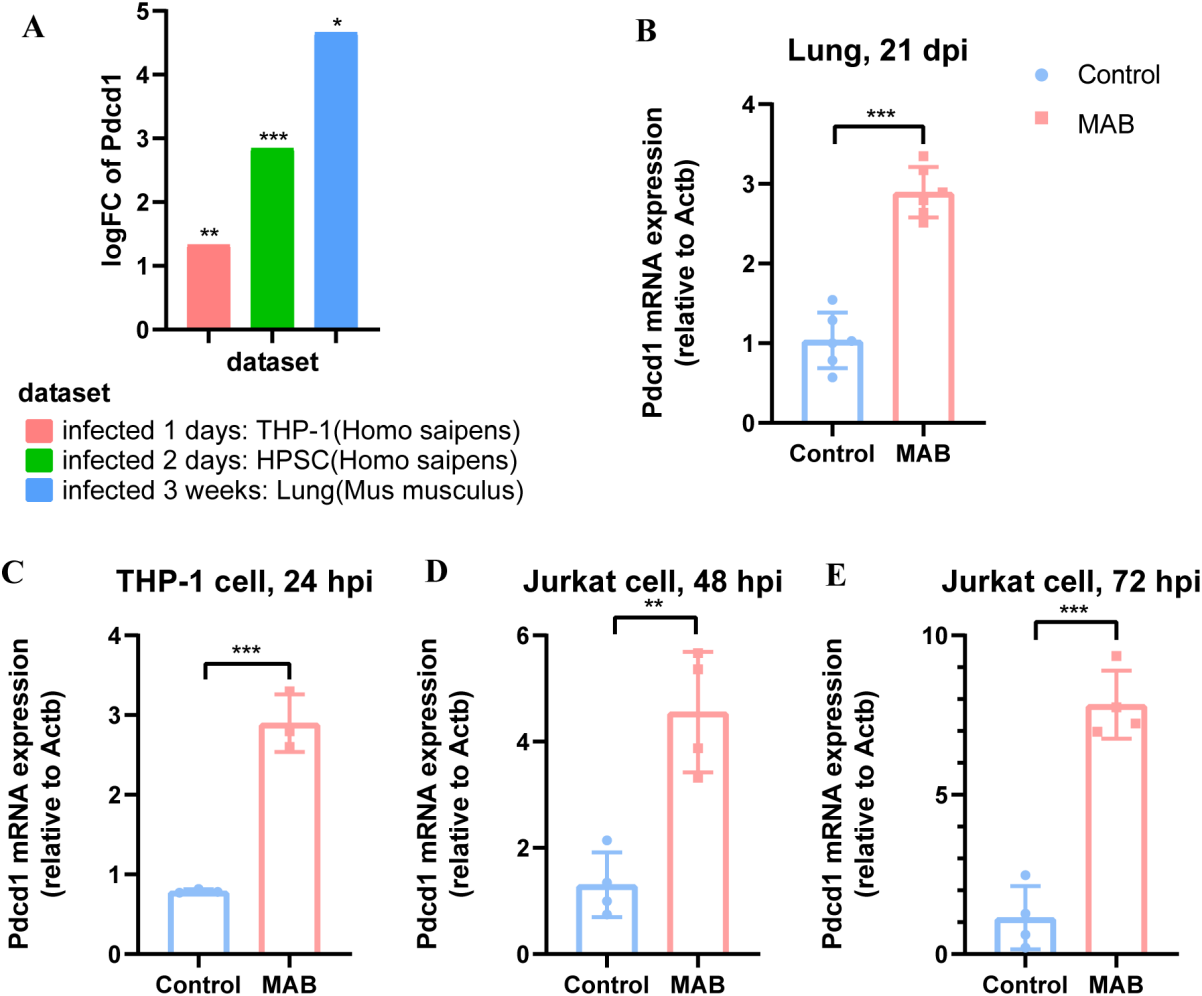
PD-1 upregulation in host cells during MAB infection. **(A)** Transcriptomic analysis of *Pdcd1* in immune cells and mouse lung tissues after MAB infection. The control groups from left to right are: uninfected THP-1 cells, uninfected human pluripotent stem cell-derived macrophages, and lung from mice treated with saline. **(B)** RT-PCR analysis of *Pdcd1* expression in mouse lungs at 21 days post infection (dpi) with MAB (n=6 per group). **(C–E)** *Pdcd1* expression in THP-1 cells and Jurkat cells assessed by RT-PCR at indicated time point after MAB infection (n=3–5 per group). All experiments were performed in triplicate. The differences between groups were calculated using an unpaired t-test. Data are shown as mean ± SD. *P < 0.05, **P < 0.01, ***P < 0.001, ns means no significance.

To investigate the potential role of PD-1 in the host response to MAB infection, we analyzed published transcriptomic data(GSE140438, GSE207456, GSE72822), revealing upregulated *Pdcd1* expression in mouse lung, human macrophages and human pluripotent stem cell-derived macrophages (**Figure 1A**). RT-PCR confirmed this upregulation in MAB-infected lung of mice (**Figure 1B**), macrophages (**Figure 1C**), and T cells (**Figures 1D, E**), implicating PD-1 in both innate and adaptive immune responses.

**Figure 2:**
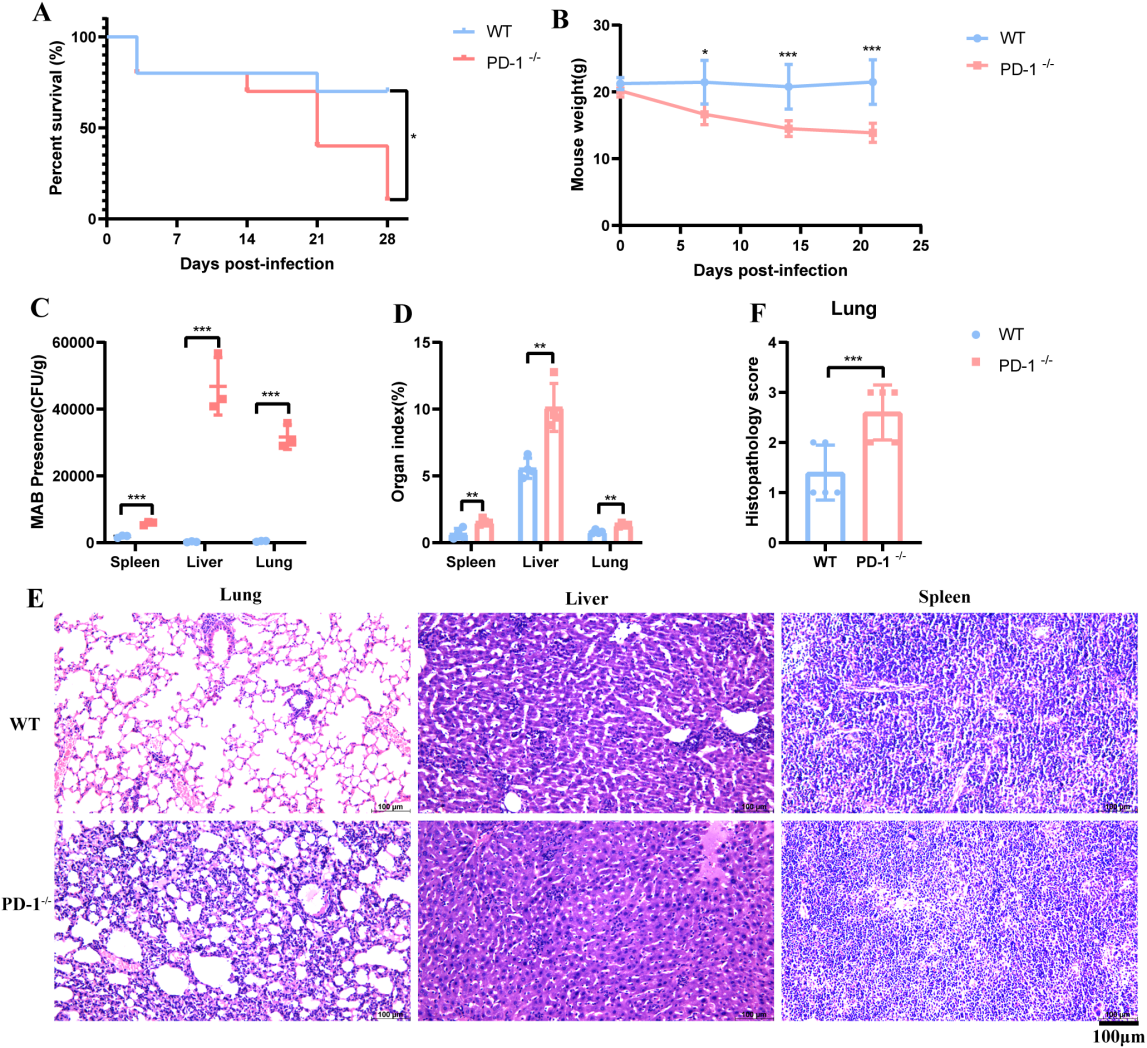
PD-1^−/−^ mice are highly susceptible to MAB infection. **(A)** Survival of WT or PD-1 deficient mice following intravenous challenge with 2×10^7^ CFU of MAB (n=10 per group). Survival curves were compared by the log-rank test. **(B)** Body weight changes of WT and PD-1 deficient mice after MAB infection (n=6 per group). The differences between groups were calculated using a log-rank test. **(C)** Bacterial loads in the spleen, liver, and lungs of WT and PD-1 deficient mice at 21 days post-infection (n=3 per group). **(D)** Organ indices of the spleen, liver, and lungs in WT and PD-1 deficient mice at 21 days post-infection (n=5 per group). **(E)** Representative hematoxylin and eosin–stained tissue sections at 21 days post-infection (n=4 per group). Scale bar, 100 µm. **(F)** Clinical scores were assessed using a previously established scoring system for murine bacterial infection models (n=5 per group). All experiments were performed in triplicate. The differences between groups were calculated using an unpaired t-test. Data are shown as mean ± SD. *P < 0.05, **P < 0.01, ***P < 0.001, ns means no significance.

To further elucidate the role of PD-1 in host defense against MAB infection, we employed MAB infected PD-1^−/−^ and WT mouse models. PD-1^−/−^ mice exhibited markedly increased mortality by day 21 post-infection (**Figure 2A**), accompanied by significant weight loss (**Figure 2B**). By day 28, nearly all PD-1^−/−^ mice had succumbed, while most WT mice survived. Assessment of bacterial loads revealed significantly higher burdens in the liver, spleen, and lungs of PD-1^−/−^ mice at 21 days (**Figure 2C**), whereas no differences were observed at earlier time points (days 3 and 7; data not shown).

In MAB-infected PD-1^−/−^ mice, marked hepatosplenomegaly and pneumomegaly were observed. Organ indices for the liver, spleen, and lung were significantly elevated in PD-1^−/−^ mice compared to WT controls (**Figure 2D**). PD-1 deficiency exacerbated organ damage, most severely in the lungs (**Figure 2E**), as indicated by pathological evaluation and corroborated by lung histopathological scores (**Figure 2F**). PD-1^−/−^ mice displayed extensive inflammatory cell infiltration in alveolar septa, in contrast to minimal infiltration in WT mice. Inflammatory infiltration in the liver and spleen was similar between groups.

Collectively, our findings show that PD-1^−/−^ mice are more susceptible to MAB infection and exhibit impaired control of hematogenous dissemination, underscoring the critical role of PD-1 in containing MAB infection.

### 3.2 During MAB infection, PD-1 deficiency impaired the recruitment of immune cells within lung

To examine the effect of PD-1 deficiency on immune cell recruitment in mouse spleen and lungs, we analyzed immune cell composition in WT and PD-1^−/−^ mice. No significant differences were found (**Supplementary Figure 2**), indicating comparable baseline states. Thus, post-infection lung immune cell infiltration differences were MAB-induced.

**Figure 3:**
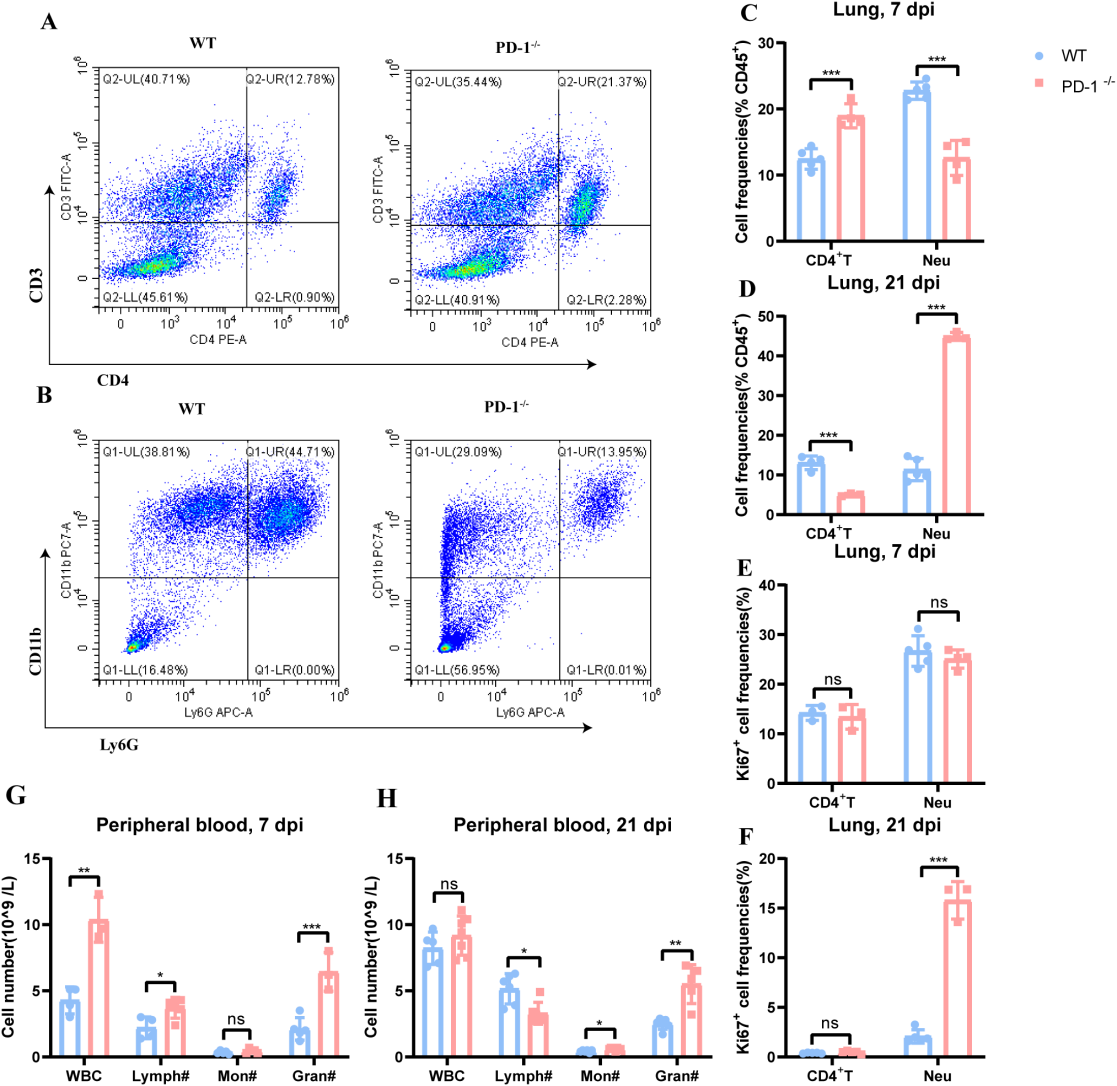
Impaired recruitment of inflammatory cells in PD-1-deficient mice during MAB infection. **(A, B)** Representative flow cytometry plots of CD4^+^ T cells and neutrophils in lung from WT and PD-1^−/−^ mice at 7 days post-MAB infection, (n = 3–5 per group). **(C, D)** Proportions of CD4^+^ T cells and neutrophils in the lungs of WT and PD-1^−/−^ mice at 7 and 21 days post-MAB infection, (n = 3–5 per group). **(E, F)** Representative Ki-67 expression in CD4^+^ T cells and neutrophils from the lungs of WT and PD-1^−/−^ mice at 7 and 21 days post-MAB infection, (n = 3–5 per group). **(G, H)** Cell counts in peripheral blood samples from WT and PD-1^−/−^ mice at 7 and 21 days post-MAB challenge, (n = 5–8 per group). All experiments were performed in triplicate. The differences between groups were calculated using an unpaired t-test. Data are shown as mean ± SD. *P < 0.05, **P < 0.01, ***P < 0.001, ns means no significance.

To elucidate how PD-1 deficiency enhances MAB infection susceptibility in mice, we developed a murine model and evaluated immune cell profile in peripheral blood and lung tissues at days 7 and 21 post-infection. In the early phase (7 days), PD-1^−/−^ mice showed increased proportions of CD4^+^ T cells and decreased neutrophils in lung (**Figures 3A–C**), with no other immune cell differences (**Supplementary Figures 3A, B**). Peripheral blood displayed elevated lymphocyte and neutrophil counts (**Figure 3G**). During the late phase(21 days), PD-1^−/−^ mice had reduced CD4^+^ T cells and increased neutrophils in lung (**Figure 3D**). Proportions of alveolar macrophages(AM), natural killer(NK) cells, dendritic cells (DCs) and CD8^+^ T cells decrease (**Supplementary Figures 3C, D**). The elevated pulmonary neutrophils may result from enhanced proliferative capacity, as evidenced by increased Ki67 expression (**Figure 3F**). In blood, lymphocyte counts declined, whereas neutrophils increased (**Figure 3H**).

In summary, PD-1 deficiency impairs the recruitment of inflammatory cells to the site of infection, consequently increasing the susceptibility of mice to MAB.

### 3.3 PD-1 deficiency affects T cell activation and neutrophil extracellular trap formation

**Figure 4:**
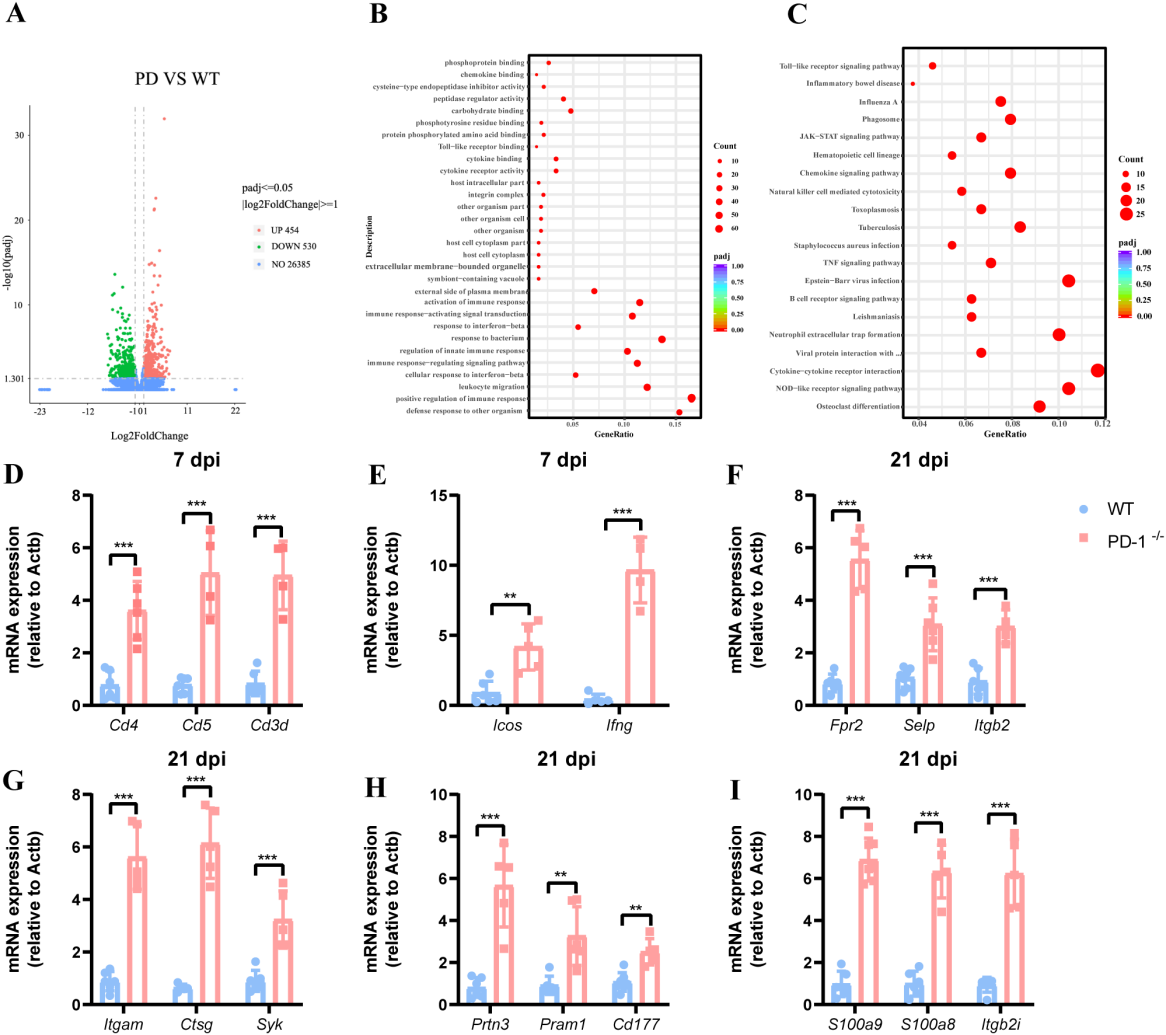
PD-1 deficiency alters expression of considerable genes involved in innate and adaptive immunity during MAB infection. **(A)** Volcano plot depicting differentially expressed genes from RNA-seq in the lung of WT and PD-1^−/−^ mice challenged with MAB at 21 day. Significantly upregulated and downregulated genes are highlighted in red and green, respectively. Values indicate log_2_ fold change, (n = 5 per group). **(B)** Gene Ontology (GO) enrichment analysis of biological processes for upregulated genes, (n = 5 per group). **(C)** KEGG pathway enrichment analysis of upregulated targets in the PD-1-deficient transcriptomes, (n = 5 per group). **(D–I)** qPCR validation of selected PD-1 modulated genes in mouse lungs at 7 and 21 days post-MAB infection, (n = 5–7 per group). All experiments were performed in triplicate. The differences between groups were calculated using an unpaired t-test. Data are shown as mean ± SD. *P < 0.05, **P < 0.01, ***P < 0.001, ns means no significance.

Transcriptomic analysis of lungs from WT and PD-1^−/−^ mice at 7 and 21 days post-MAB infection revealed distinct immune profiles. At day 7, 378 differentially expressed genes (DEGs) were identified in PD-1^−/−^ mice, with 233 upregulated and 125 downregulated (**Supplementary Figure 4A**). Upregulated genes were enriched in immune-related Gene Ontology (GO) terms and Kyoto Encyclopedia of Genes and Genomes (KEGG) pathways, including T cell receptor signaling, Th1 and Th2 cell differentiation, and Th17 cell differentiation (**Supplementary Figures 4B, C**). By day 21, 984 DEGs were detected—454 upregulated and 530 downregulated (**Figure 4A**). GO analysis highlighted processes such as leukocyte migration and defense response (**Figure 4B**), while KEGG analysis indicated upregulation in chemokine signaling, neutrophil extracellular trap formation, and cytokine–cytokine receptor interaction (**Figure 4C**). These findings suggest PD-1 deficiency amplifies both adaptive and innate immune responses over the course of infection.

We validated hub genes linked to the T cell receptor signaling pathway and neutrophils activation by RT-PCR. In PD-1-deficient mice, lung expression of *Cd3d*, *Cd4*, *Cd5*, *Ifng*, and *Icos* was significantly upregulated at 7 dpi (**Figures 4D, E**). Moreover, PD-1 deficiency further enhanced *S100a8*, *S100a9*, *Itgam*, *Ctsg*, *Syk* and *Itgb2* expression by day 21 (**Figures 4F–I**).

In summary, PD-1 deficiency upregulates key immune pathway genes during infection: early stage involves T cell receptor signaling, while later stages show neutrophil activation and NETs pathways, which are vital for host defense against MAB.

### 3.4 PD-1 deficiency impairs the balance of CD4^+^ T cell subsets

**Figure 5:**
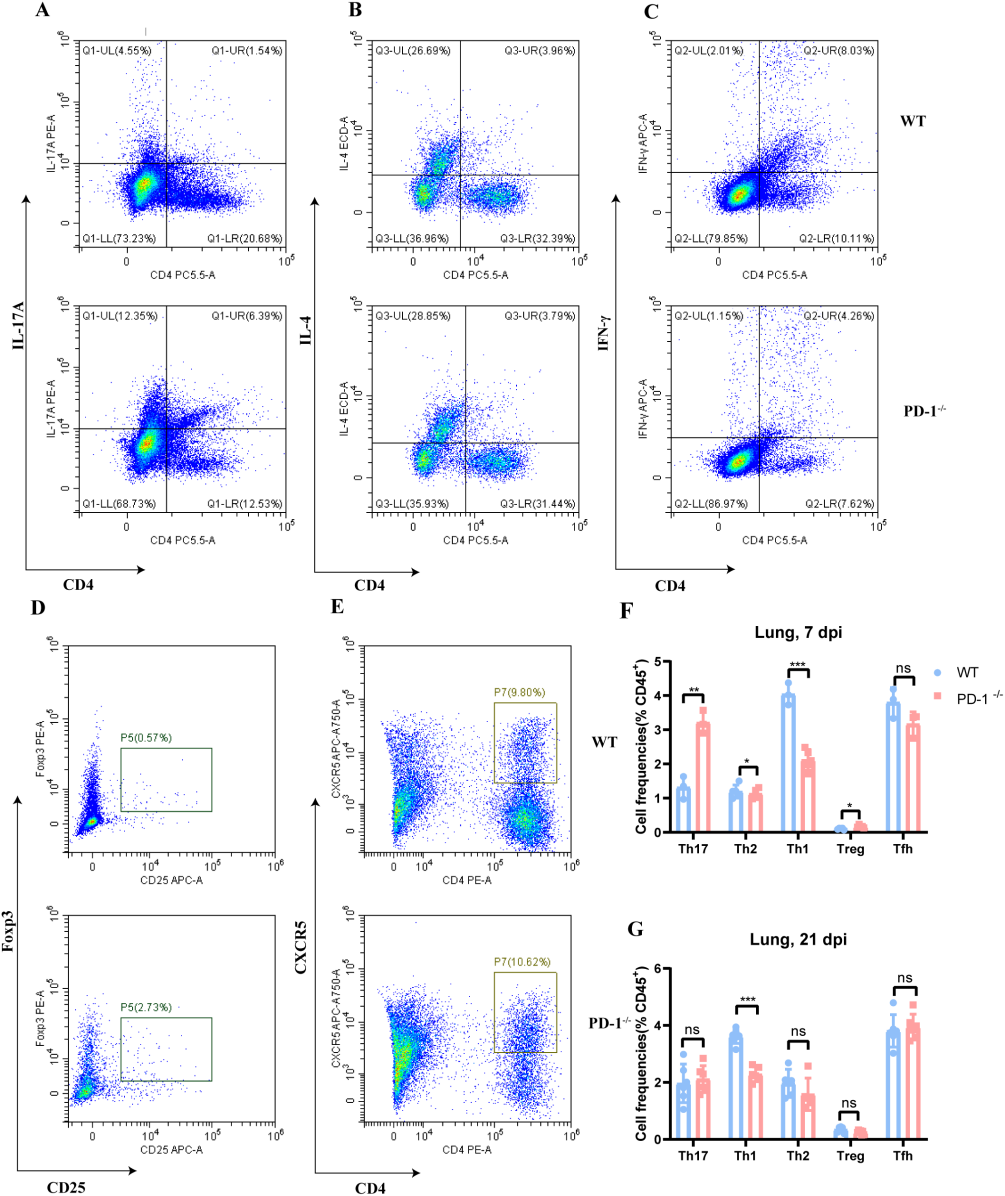
PD-1 deficiency disrupts CD4^+^ T cell polarization following MAB infection. **(A–E)** Representative flow plots of Th17, Th2, Th1, Treg, and Tfh cells within CD45^+^ cells from WT and PD-1^−/−^ mice at 7 days after MAB infection. **(F)** Quantification of the cell populations shown in **(A–E)**, (n = 3–5 per group). **(G)** Frequencies of Th17, Th2, Th1, Treg, and Tfh cells among CD45^+^ cells in lung of WT and PD-1^−/−^ mice at 21 days post-infection, (n = 6–9 per group). All experiments were performed in triplicate. The differences between groups were calculated using an unpaired t-test. Data are shown as mean ± SD. *P < 0.05, **P < 0.01, ***P < 0.001, ns means no significance.

The immune response presents a double-edged sword, where dysregulated T-cell activation or subset imbalance can trigger inflammation, allergy, or autoimmunity. Transcriptomic profiling indicated that PD-1 deficiency in MAB-infected mice disrupted Th1, Th2, and Th17 differentiation and T-cell receptor signaling. We thus assessed CD4^+^ T cell subsets in lungs at 7 and 21 days post-infection. At day 7, PD-1^−/−^ mice showed elevated Th17 (**Figures 5A, F**) and Treg (**Figures 5D, F**) populations in the lungs, alongside reduced Th1(**Figures 5C, F**) cells. By day 21, Th1 cells further declined, with no significant changes in other subsets (**Figure 5G**). Overall, PD-1 deficiency skewed CD4^+^ T cell balance in the lung following MAB infection.

### 3.5 PD-1 deficiency affects antimicrobial function of neutrophils

Neutrophils combat pathogens through mechanisms including ROS production, enzyme release (e.g., NE, MPO), cytokine secretion and NETs formation (5). To assess PD-1’s role, we evaluated phagocytosis and ROS in bone marrow-derived neutrophils from WT and PD-1^−/−^ mice, observing no significant differences (data not shown).

**Figure 6:**
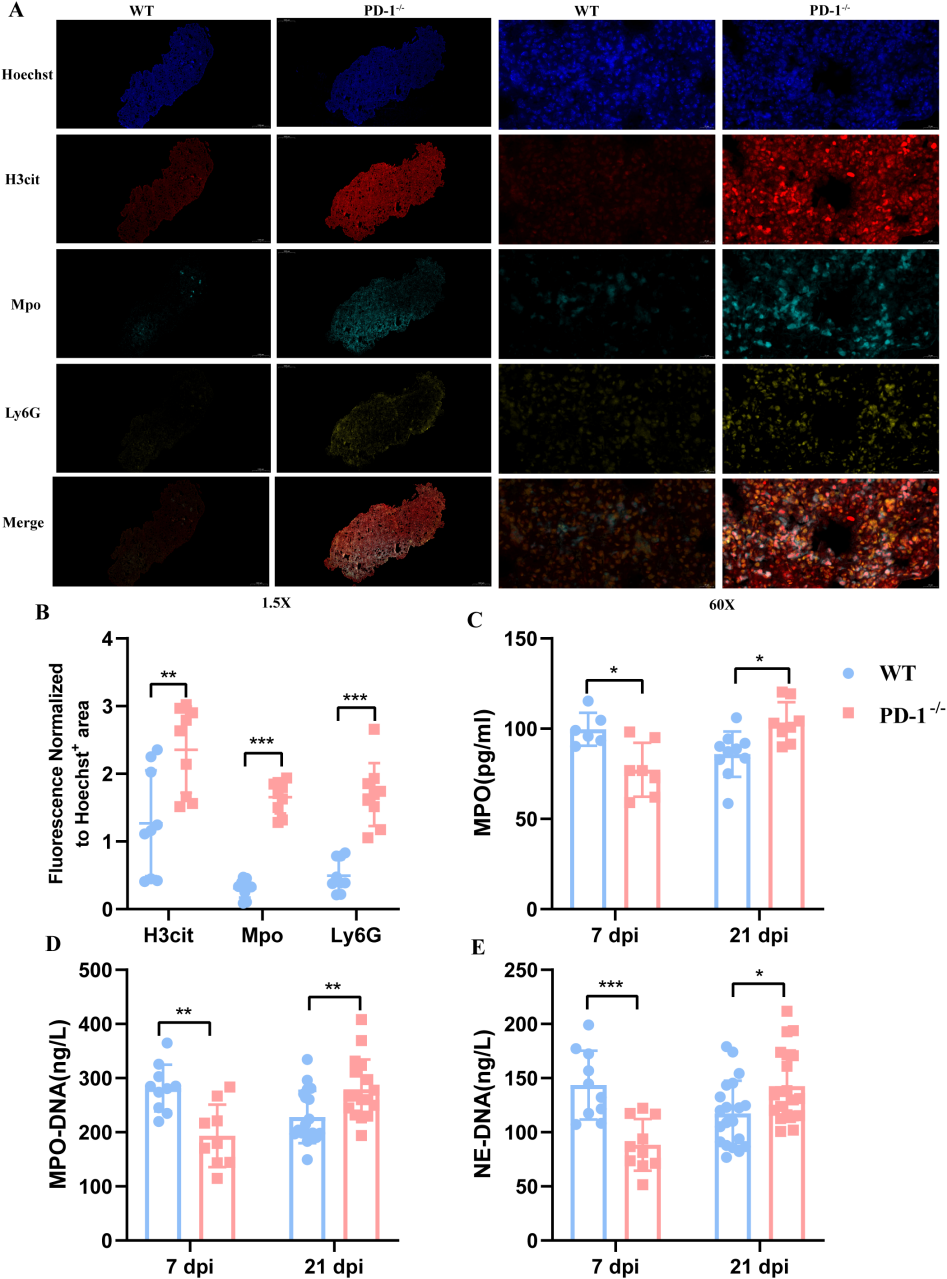
PD-1 deficiency enhances neutrophil extracellular trap formation in peripheral blood and lung of mice following MAB challenging. **(A)** Representative immunofluorescence images of lung sections from MAB-infected WT and PD-1^−/−^ mice stained with antibodies against citrullinated histone H3 (H3Cit, red), myeloperoxidase (MPO, teal), Ly6G (yellow), and DNA (Hoechst, blue) at 21 day. **(B)** Quantification of H3Cit, MPO, and Ly6G fluorescence normalized to the total Hoechst-positive area in lung sections of WT and PD-1^−/−^ mice at 21 days post-infection, (n = 3–5 per group). **(C–E)** MPO, NE-DNA and MPO-DNA concentrations in peripheral blood from WT and PD-1^−/−^ mice were measured by ELISA at 7 days (n = 10 per group) and 21 days post-infection, (n = 20 per group). All experiments were performed in triplicate. The differences between groups were calculated using an unpaired t-test. Data are shown as mean ± SD. *P < 0.05, **P < 0.01, ***P < 0.001, ns means no significance.

We next examined protease release and NET formation in peripheral blood at 7 and 21 days post-MAB infection. Early during infection, PD-1^−/−^ mice exhibited markedly reduced levels of MPO, MPO–DNA, and NE–DNA compared to WT mice (**Figures 6C–E**). In contrast, by day 21, these markers were significantly elevated in PD-1^−/−^ mice. Additionally, immunofluorescence staining for the NET marker H3cit in infected lungs showed comparable early expression between genotypes(data not shown), but significantly increased levels in PD-1^−/−^ mice at later stages (**Figures 6A, B**).

Together, these results indicate that PD-1 deficiency not only impairs neutrophil recruitment but also disrupts their antimicrobial functions over the course of infection.

### 3.6 PD-1 deficiency alters production of cytokine and chemokine

**Figure 7:**
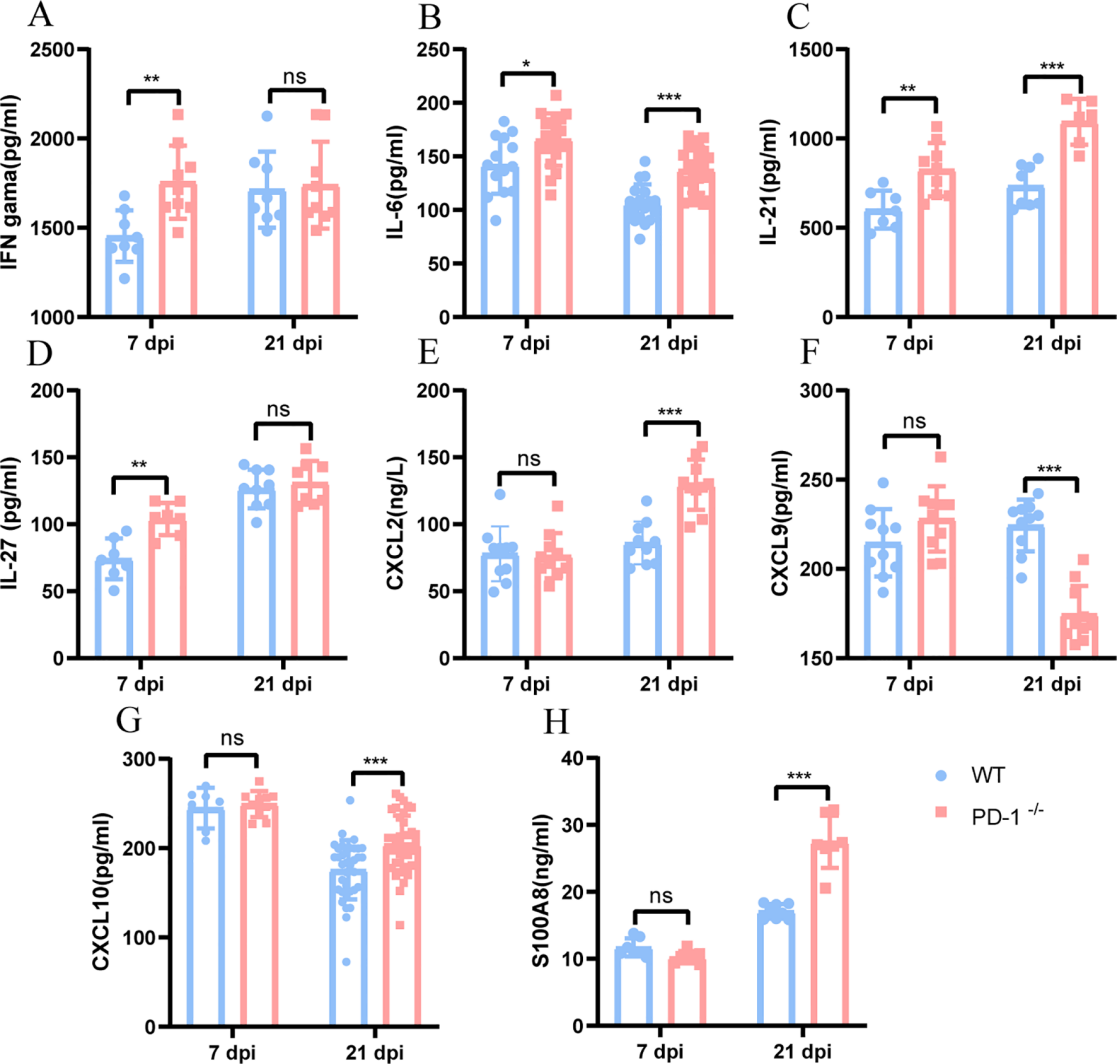
PD-1 deficiency leads to extensive change of cytokine and chemokine profile in peripheral blood of mice challenged with MAB. **(A–H)** IFN-γ, IL-6, IL-21, IL-27, CXCL2, CXCL9, CXCL10, and S100A8 concentrations in peripheral blood from WT and PD-1^−/−^ mice were quantified after MAB infection at day 7 and day 21 by ELISA, (n = 7–40 per group). All experiments were performed in triplicate. The differences between groups were calculated using an unpaired t-test. Data are shown as mean ± SD. *P < 0.05, **P < 0.01, ***P < 0.001, ns means no significance.

During bacterial infection, cytokines and chemokines act in concert to orchestrate the recruitment of immune cells, synergistically guiding them in a precisely coordinated manner to the site of infection. We evaluated serum cytokine and chemokine profiles in WT and PD-1^−/−^ mice after MAB infection. At day 7, PD-1^−/−^ mice had increased IFN-γ, IL-6, IL-21, and IL-27 (**Figures 7A–D**), but no change in CXCL2, CXCL9, CXCL10, or S100A8 (**Figures 7E–H**). By day 21, IL-6, IL-21, CXCL2, CXCL10, and S100A8 were elevated (**Figures 7B–H**), while CXCL9 was decreased (**Figure 7F**), IFN-γ and IL-27 remained comparable (**Figures 7A, B**).

### 3.7 PD-1 deficiency leads to activation of the HIF-1 signaling pathway and metabolic reprogramming

**Figure 8:**
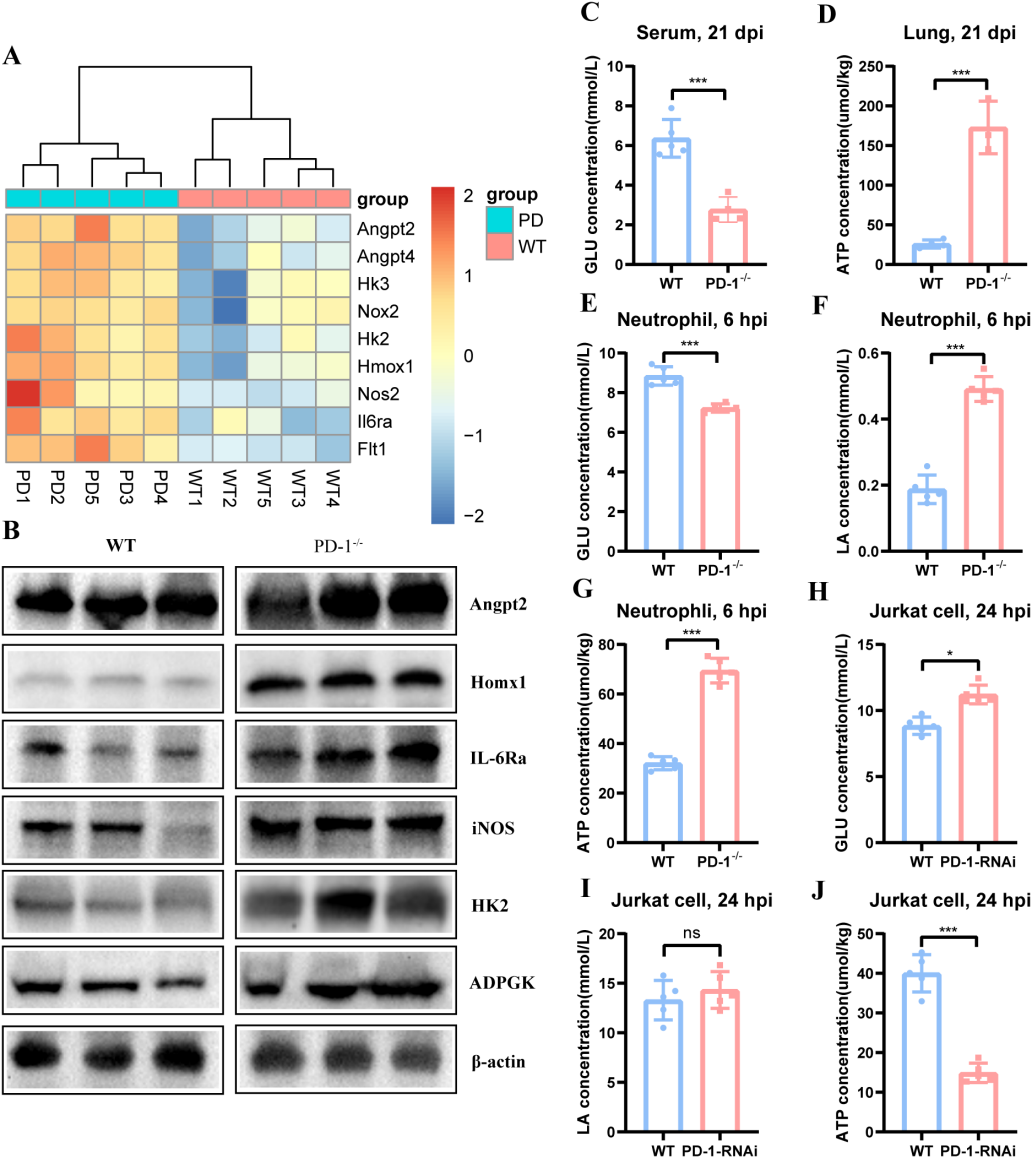
PD-1 deficiency induces HIF-1 signaling activation in mouse lung and mediates the metabolic reprogramming in mice, Jurkat cells and neutrophils after MAB infection. **(A)** HIF-1 signaling pathway heatmap in the lung of WT and PD-1^−/−^ mice at 21 dpi with MAB, (n = 3–5 per group). **(B)** Representative western blots of HIF-1 signaling pathway proteins in lung tissues from MAB-infected WT and PD-1^−/−^ mice at 21 dpi with MAB, (n = 3 per group). **(C, D)** Serum glucose levels and pulmonary ATP concentrations in WT and PD-1^−/−^ mice at 21 dpi with MAB, (n = 3–5 per group). **(E–G)** Supernatant glucose and lactate, and cellular ATP in WT and PD-1^−/−^ neutrophils post 6 h MAB infection, (n = 3–5 per group). **(H–J)** Supernatant glucose and lactate, and cellular ATP in WT and PD-1 knockdown Jurkat cell following 24 h MAB infection, (n = 3–5 per group). All experiments were performed in triplicate. The differences between groups were calculated using an unpaired t-test. Data are shown as mean ± SD. *P < 0.05, **P < 0.01, ***P < 0.001, ns means no significance.

HIF-1, a heterodimeric transcription factor (HIF-1α/β), mediates hypoxia adaptation by binding HREs to activate genes including EPO and VEGF, thereby regulating glucose metabolism, mitochondrial function, apoptosis, and antioxidant responses (15, 16). Previous data indicated PD-1 deficiency upregulates the HIF-1 pathway (**Figure 8A**). Western blot analysis showed increased expression of glycolytic regulators Hk2, angiogenesis-associated Angpt2, oxidative stress-related Hmox1and iNOS, and inflammatory mediator Il6ra (**Figure 8B**). Late in infection, PD-1^−/−^ mice displayed reduced blood glucose (**Figure 8C**) and elevated lung ATP synthesis (**Figure 8D**) and, with minimal glucose and lactate in lung tissue (**Supplementary Figures 5D, E**). ADPGK expression was also heightened. *In vitro*, PD-1 deficiency enhances glucose consumption and ATP/lactate synthesis in neutrophils (**Figures 8E–G**) but reduces glucose consumption and ATP synthesis in Jurkat cells (**Figures 8H, J**), with lactate production unaffected in Jurkat cells after MAB infection (**Figure 8I**).

**Figure 9:**
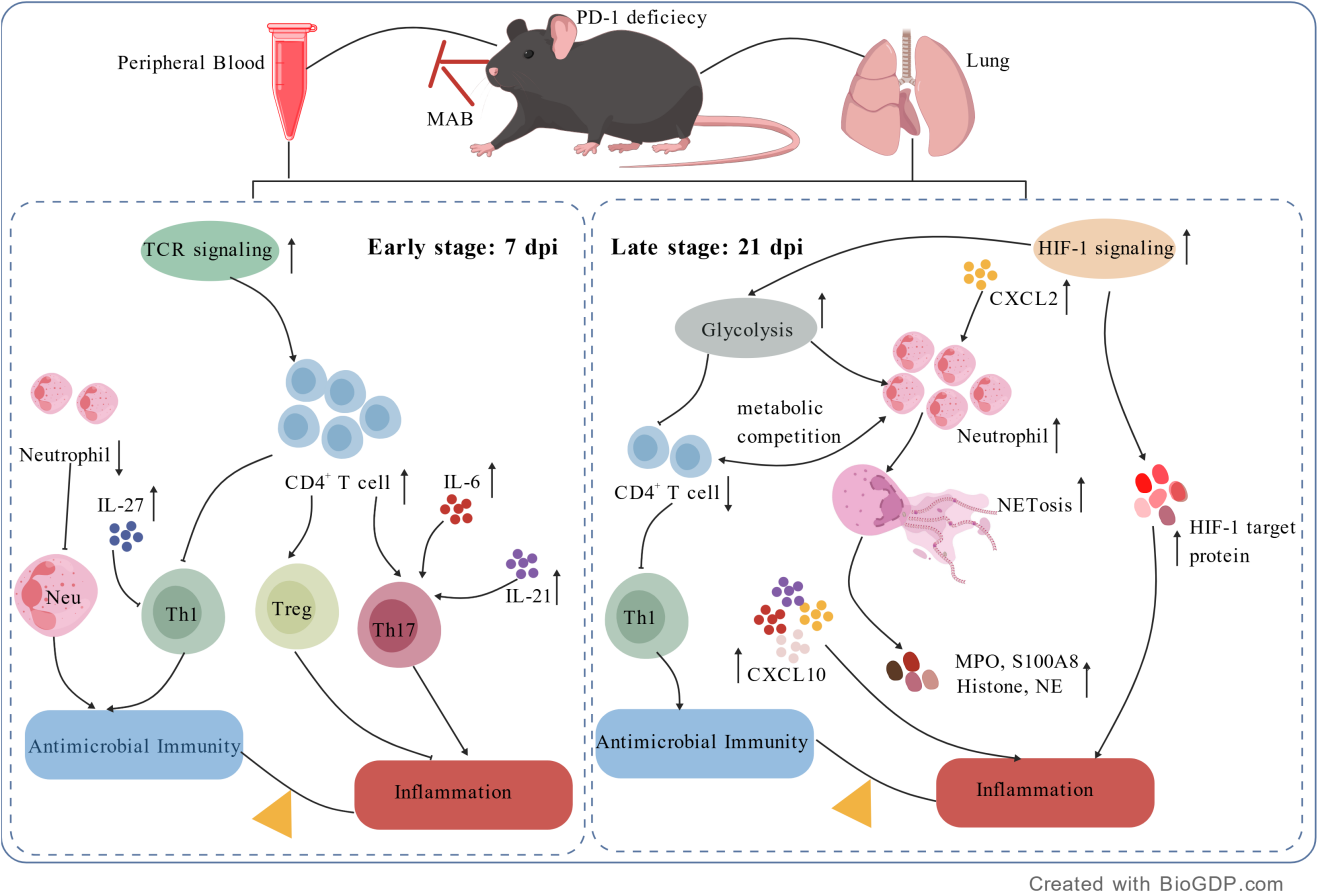
The possible mechanisms of PD-1 deficiency in exacerbating MAB infection. PD-1 deficiency disrupts TCR signaling, leading to imbalance among Th1 (anti-infection), Th17 (pro-inflammatory), and Treg (immunosuppressive) responses. Neutrophils exhibit impaired early recruitment and antimicrobial activity, followed by excessive NETosis and tissue injury. HIF-1 activation enhances glycolysis, elevating pulmonary ATP and upregulating HK2 and iNOS, reflecting a Warburg-like effect. Systemic cytokine/chemokine dysregulation, including increased IL-6, IL-21 and CXCL10, perpetuates chronic inflammation.

These results demonstrate that PD-1 deficiency drives metabolic reprogramming of T cell and neutrophils in mice after MAB infection (**Figure 9**).

## 4 Discussion

### 4.1 Results and significance

In a PD-1-deficient mouse model, this study establishes the essential protective function of the PD-1 pathway in host defense against MAB infection, offering novel perspectives on NTM immunopathology. PD-1 deficiency severely compromises the host’s capacity to clear MAB, primarily through immune-metabolic dysregulation throughout infection. Initially, increased CD4^+^ T cell infiltration and neutropenia are observed, which reverses later, disrupting Th1/Th17/Treg homeostasis and weakening antibacterial responses. This imbalance also causes aberrant neutrophil recruitment and impaired NETosis, shifting from antimicrobial to pro-inflammatory and tissue-damaging phenotypes. Dysregulated cytokine/chemokine networks exacerbate immune cell mislocalization and dysfunction. Furthermore, activation of the HIF-1 signaling pathway and glycolysis-driven metabolic reprogramming in the lung microenvironment create a vicious immune-metabolic cycle that undermines host defenses. Consequently, PD-1/PD-L1 inhibitors may increase the risk of NTM infections, particularly MAB, in cancer patients, as supported by clinical report (12), providing crucial insights for management.

### 4.2 The impact of PD-1 deficiency on T cell immunity

In PD-1^−/−^ mice, MTB infection elevates the frequency of MTB-specific CD4^+^ T cells (17), resembling early MAB infection. However, PD-1 deficiency compromises Th1 immunity against MAB, impairing bacterial control. During MTB infection, macrophage-derived IL-12 drives Th1 differentiation and IFN-γ production, activating macrophages to clear pathogens. IFN-γ further sustains IL-12 secretion, creating a positive feedback loop that amplifies Th1 responses and IFN-γ release (18). Although PD-1^−/−^ mice exhibit increased IFN-γ-producing CD4^+^ T cells upon ESAT-6 restimulation *in vitro*, this heightened Th1 response fails to confer effective MTB containment (17). The persistent Th1 impairment due to PD-1 deficiency likely explains the host’s inability to control MAB.

Single-cell studies distinguish pathogenic from non-pathogenic Th17 subsets: both secrete IL-17, but non-pathogenic Th17 cells co-express immunoregulatory genes like Il10, Il9, Maf, and Ahr. Pathogenic Th17 cells, however, produce pro-inflammatory factors such as Csf2, Ifng, Tbx21, IL23R, and GZMB (19, 20), and exacerbate pathology in autoimmune models (21, 22). In tuberculosis, Th17 cells typically protect the host by secreting IL-17A, IL-17F, IL-21, and IL-22, which induce antimicrobial peptides and recruit phagocytes (23). However, in our MAB model, elevated pulmonary Th17 cells likely recruited neutrophils during late infection, aggravating tissue injury and chronic inflammation without aiding bacterial clearance—indicating a pathogenic role.

Tregs, as immunomodulatory cells, suppress Th1, Th2, and Th17 effector functions and limit inflammation via TGF-β and IL-10 (24). In active tuberculosis patients, elevated Treg levels negatively correlate with PD-1/PD-L1 expression, and their downregulation promotes Treg proliferation (25). Accordingly, PD-1 deficiency during MAB infection increases Treg frequency, which may further suppress the compromised Th1 response and exacerbate bacterial dissemination. Thus, PD-1 deficiency disrupts the balance among protective Th1, inflammatory Th17, and immunosuppressive Treg responses, undermining anti-MAB immunity.

### 4.3 The impact of PD-1 deficiency on innate immunity (neutrophils)

NETs are web-like structures composed of DNA filaments and antimicrobial proteins—including citrullinated histones, defensins, neutrophil elastase (NE), and myeloperoxidase (MPO)—that trap and neutralize pathogens, thereby containing infection and limiting microbial dissemination (26). In this study, PD-1-deficient mice exhibited enhanced NETs formation upon MAB infection, yet paradoxically displayed higher bacterial loads, suggesting impaired pathogen clearance. This discrepancy may reflect the context-dependent efficacy of NETs, as their composition critically influences antimicrobial activity. For example, cathepsin G deficiency reduces NET-mediated killing of *Staphylococcus aureus (*27), and certain trapped bacteria, such as *Pseudomonas aeruginosa*, can remain viable and even resist complement-mediated killing (28). Furthermore, excessive NET-associated components like histones, MPO, and NE can exacerbate pulmonary inflammation and disrupt immune homeostasis (29).

Notably, PD-1-deficient neutrophils showed no intrinsic defects in phagocytosis or ROS production, implying that elevated NET release and upregulated Nox2 and iNOS expression likely stem from systemic immune dysregulation—such as altered T-cell responses and cytokine storms—triggered by PD-1 loss.

In summary, PD-1 signaling regulates neutrophil recruitment via the CXCL2 and fine-tunes NET generation. Excessive NETosis in the absence of PD-1 compromises antibacterial defense during MAB infection, underscoring the importance of NET homeostasis in effective immunity.

### 4.4 Cytokine and chemokine dysregulation in PD-1 deficiency

PD-1 deficiency disrupts the coordinated cytokine and chemokine response during infection, resulting in a biphasic dysregulation that promotes immunopathology.

During early infection, an aberrant cytokine profile emerged, marked by elevated IFN-γ—mainly from NK cells—that contributed to systemic inflammation without effectively activating macrophages. Increased IL-6 and IL-21 synergistically promoted pathogenic Th17 differentiation, while elevated IL-27 likely impaired Th1 immunity.

In the late phase, the response shifted toward sustained inflammation and neutrophil-driven injury. Persistently high IL-6 and IL-21 exacerbated lung damage, while elevated CXCL2 promoted robust neutrophil infiltration. This was associated with NET formation and release of the inflammatory mediator S100A8. Chemokine dysregulation was also evident: reduced CXCL9 limited CD8^+^ T cell recruitment, whereas elevated CXCL10 exacerbated pulmonary inflammation.

In summary, PD-1 is essential for immune equilibrium. Its absence initiates a maladaptive early inflammatory response that progresses to a late phase dominated by neutrophil hyperactivation and defective adaptive immunity, culminating in tissue injury.

### 4.5 HIF-1 signaling and metabolic reprogramming

Our findings reveal that PD-1 deficiency elicits a pathogenic metabolic state driven by HIF-1 signaling, which exacerbates inflammatory injury and impairs host defense. In the lungs of PD-1-deficient mice at late infection stages, HIF-1 activation upregulated pro-inflammatory target genes—including Il-6ra, iNOS, and Angpt2. This response induced a glycolytic shift, characterized by increased HK2 expression and ATP production, alongside heightened glucose consumption by activated immune cells that resulted in systemic hypoglycemia.

Notably, PD-1 deficiency exerts cell-type-specific effects on glycolytic metabolism. In Jurkat T cells, it leads to reduced glycolysis, likely contributing to Th1 cell exhaustion, as optimal effector T cell responses depend on robust glycolytic activity. Conversely, PD-1 deficiency enhances glycolysis in neutrophils, fueling their hyperactivation by meeting bioenergetic and biosynthetic demands. This metabolic reprogramming creates a detrimental feedback loop: heightened glycolysis sustains innate immune hyperactivation while impairing adaptive Th1 responses through nutrient competition and inflammatory mediators. Further supporting a role for PD-1 in immunometabolic regulation, PD-1^high^ MAIT cells in tuberculous pleural effusion exhibit increased glucose uptake and lipid content, indicating that PD-1 shapes metabolic adaptations in MTB-infected MAIT cells (30).

Thus, PD-1 serves as a critical checkpoint not only for immune signals but also for immunometabolic homeostasis. Its deficiency creates a self-reinforcing network of glycolytic bias, innate immune hyperactivation, and impaired adaptive immunity, ultimately compromising microbial clearance and exacerbating tissue damage.

### 4.6 Limitations and future perspective

This study has several limitations. First, the PD-1-deficient mouse model did not fully replicate the timing, dosage, or specificity of human PD-1 inhibitor therapy. Moreover, the mouse MAB infection model differed significantly from human chronic NTM lung diseases in strain characteristics, genetic background, and underlying conditions such as cystic fibrosis or chronic obstructive pulmonary disease. Second, although phenotypes and partial mechanisms were explored, the molecular basis of PD-1 regulation—including downstream signaling effects on transcription factors and metabolic enzymes—remains incompletely elucidated. Third, the immune-metabolism relationship relied mainly on correlations, requiring direct validation via cell-specific knockouts or metabolic inhibitors. Finally, clinical data from PD-1 inhibitor users with NTM infection were absent.

Future work will clarify how PD-1 deficiency regulates T cell differentiation and neutrophil function through transcription factors and epigenetics, and its effects on HIF-1α stability and glycolytic enzymes. Using cell-specific knockouts and inhibitors, causal links between metabolic reprogramming and infection phenotypes will be established. Translational studies should assess targeting HIF-1α or glycolytic enzymes to enhance immunity, reduce pathology, and clear bacteria, exploring combination therapies.

## Data Availability

The datasets presented in this study can be found in online repositories. The names of the repository/repositories and accession number(s) can be found below: https://www.ncbi.nlm.nih.gov/, GSE317955 https://www.ncbi.nlm.nih.gov/, GSE317833.

## Supplementary material

Supplementary Figure 1 PCR genotyping of PD-1 deficient mice. PCR analysis of the PD-1 gene in wild-type (WT) and PD-1−/− mice. Lane M, DNA ladder; Lane 1, GAPDH from WT mice; Lane 2, GAPDH from PD-1−/− mice; Lane 3, PD-1 from WT mice; Lanes 4–9, PD-1 from PD-1−/− mice. Fragment sizes: mutant, ~350 bp; heterozygote, ~350 bp and 418 bp; WT, 418 bp.

Supplementary Figure 2 Representative flow cytometry gating strategy used to identify T cells, CD4+ T cells, CD8+ T cells, neutrophils, alveolar macrophages, interstitial macrophages, dendritic cells, and NK cells.

Supplementary Figure 3 Flow cytometric analysis of immune cell composition in the lung and spleen between WT and PD-1−/− mice. (A, B) Immune cell subsets (B cells, T cells, and neutrophils and etc) were quantified by flow cytometry in spleen from WT and PD-1−/− mice. (C, D) Flow cytometric evaluation of immune cell populations (B cells, T cells, and neutrophils and etc) in lung of WT and PD-1−/− mice. (n = 3 per group).

Supplementary Figure 4 Flow cytometric analysis of immune cell populations in lung of WT and PD-1−/− mice at 7 and 21 days post-MAB infection. (A, B) Frequencies of neutrophils, T cells, CD4+ T cells, CD8+ T cells, alveolar macrophages, interstitial macrophages, dendritic cells, and NK cells in lung of WT and PD-1−/− mice at 7 days post-MAB infection. (C, D) Frequencies of neutrophils, T cells, CD4+ T cells, CD8+ T cells, alveolar macrophages, interstitial macrophages, dendritic cells, and NK cells in lung of WT and PD-1−/− mice at 21 days post-MAB infection. (n = 3 per group).

Supplementary Figure 5 RNA-seq analysis of differentially expressed genes in lungs of WT and PD-1−/− mice infected with MAB for 7 days. (A) Volcano plot of differentially expressed genes from RNA-seq data comparing WT and PD-1−/− mice. Upregulated and downregulated genes are highlighted in red and green, respectively. Data are presented as log₂-transformed read counts. (B) GO enrichment analysis of biological processes for upregulated genes. (C) KEGG pathway analysis of upregulated genes in PD-1-deficient mice. (D) Hub genes identified from KEGG pathway analysis of differentially expressed genes in the lungs of WT and PD-1−/− mice after MAB infection. (n = 5 per group).

Supplementary Figure 6 Representative flow cytometry gating strategy used to identify Th1 cells, Th2 cells, Th17 cells, Treg cell, and Tfh cells.

Supplementary Figure 7 Analysis of glycolytic activity and and quantification of HIF-1 signaling pathway proteins in WT and PD-1−/− mice at 21 dpi with MAB. (A,B) Quantitative analysis of HIF-1 signaling pathway proteins expression normalized to β-actin. (C-E) Measurements of serum LA,lung LA and glucose levels in WT and PD-1−/− mice at 21 dpi following MAB infection. (F) Validation of PD-1 knockdown by RT-PCR in Jurkat cells. (n = 3-5 per group).
