# Supplementary figures and images for: PD-1 deficiency exacerbates Mycobacteroides abscessus lung infection via metabolic rewiring and dysregulated neutrophil/T cell responses
Source: Front Immunol. 2026 Apr 24;17:1803806. doi: 10.3389/fimmu.2026.1803806 (PMC13155127; doi:10.3389/fimmu.2026.1803806)

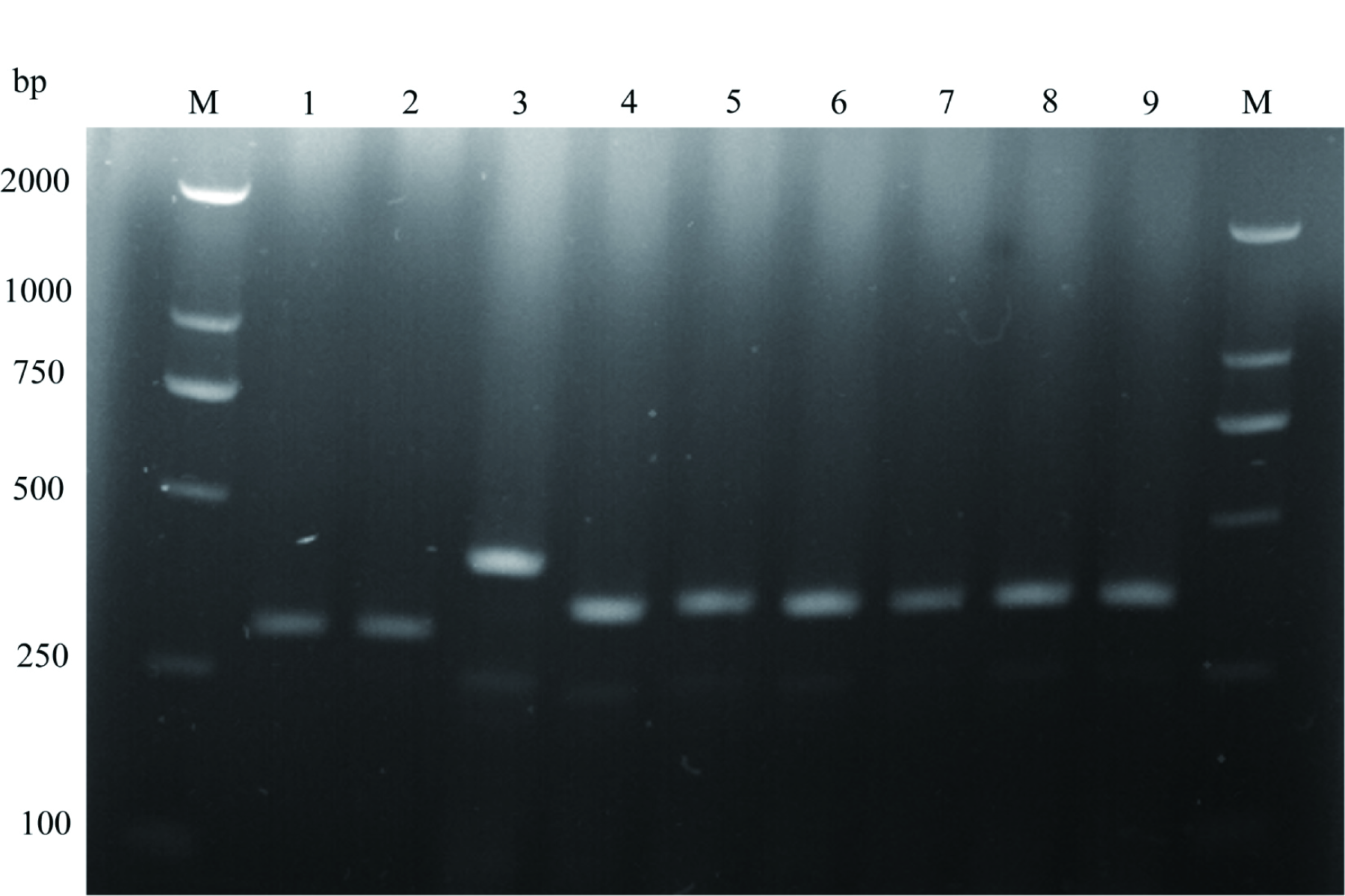

Supplement: Supplementary Figure 1 — PCR genotyping of PD-1 deficient mice. PCR analysis of the PD-1 gene in wild-type (WT) and PD-1−/− mice. Lane M, DNA ladder; Lane 1, GAPDH from WT mice; Lane 2, GAPDH from PD-1−/− mice; Lane 3, PD-1 from WT mice; Lanes 4–9, PD-1 from PD-1−/− mice. Fragment sizes: mutant, ~350 bp; heterozygote, ~350 bp and 418 bp; WT, 418 bp. [file Image1.tif]

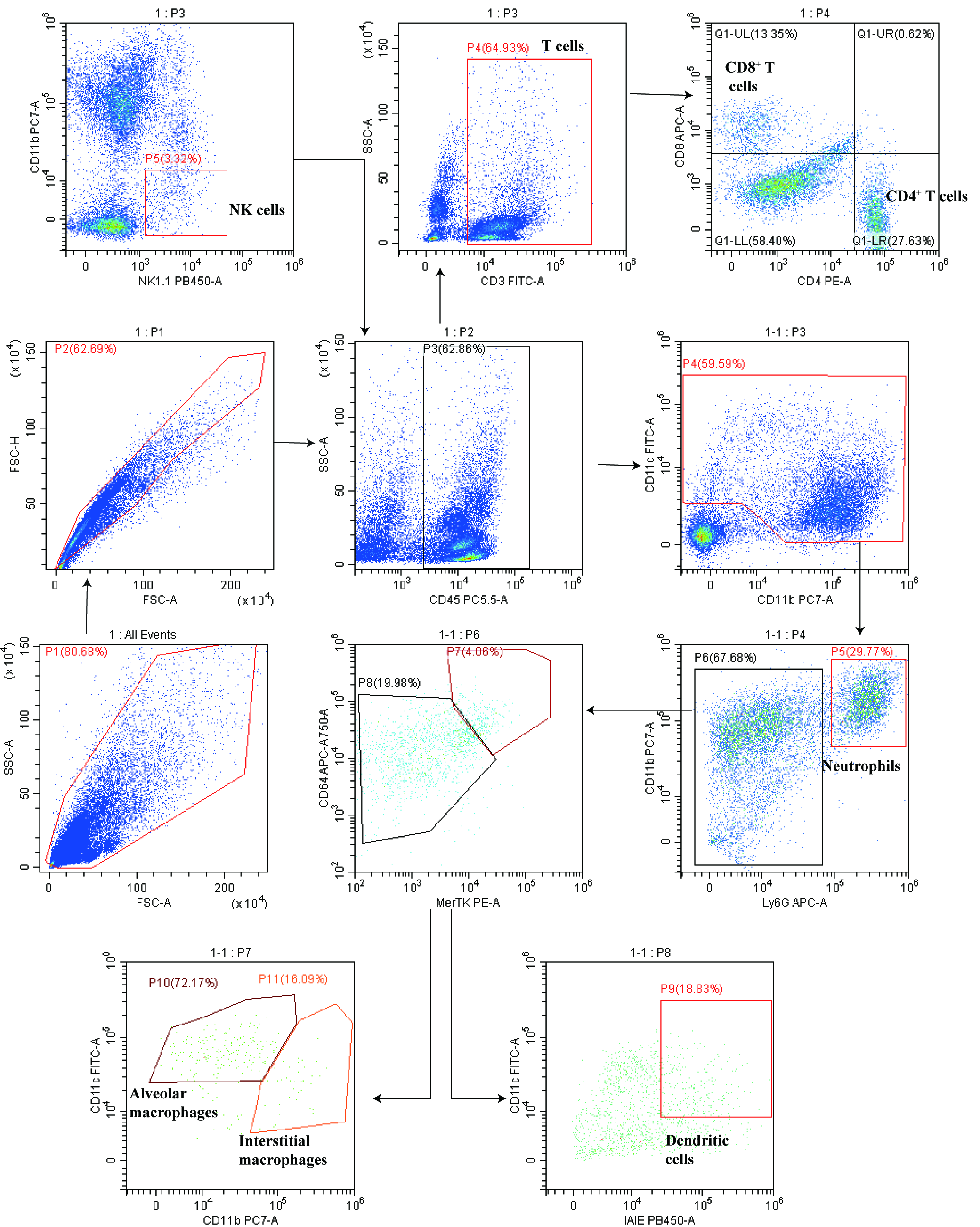

Supplement: Supplementary Figure 2 — Representative flow cytometry gating strategy used to identify T cells, CD4+ T cells, CD8+ T cells, neutrophils, alveolar macrophages, interstitial macrophages, dendritic cells, and NK cells. [file Image2.tif]

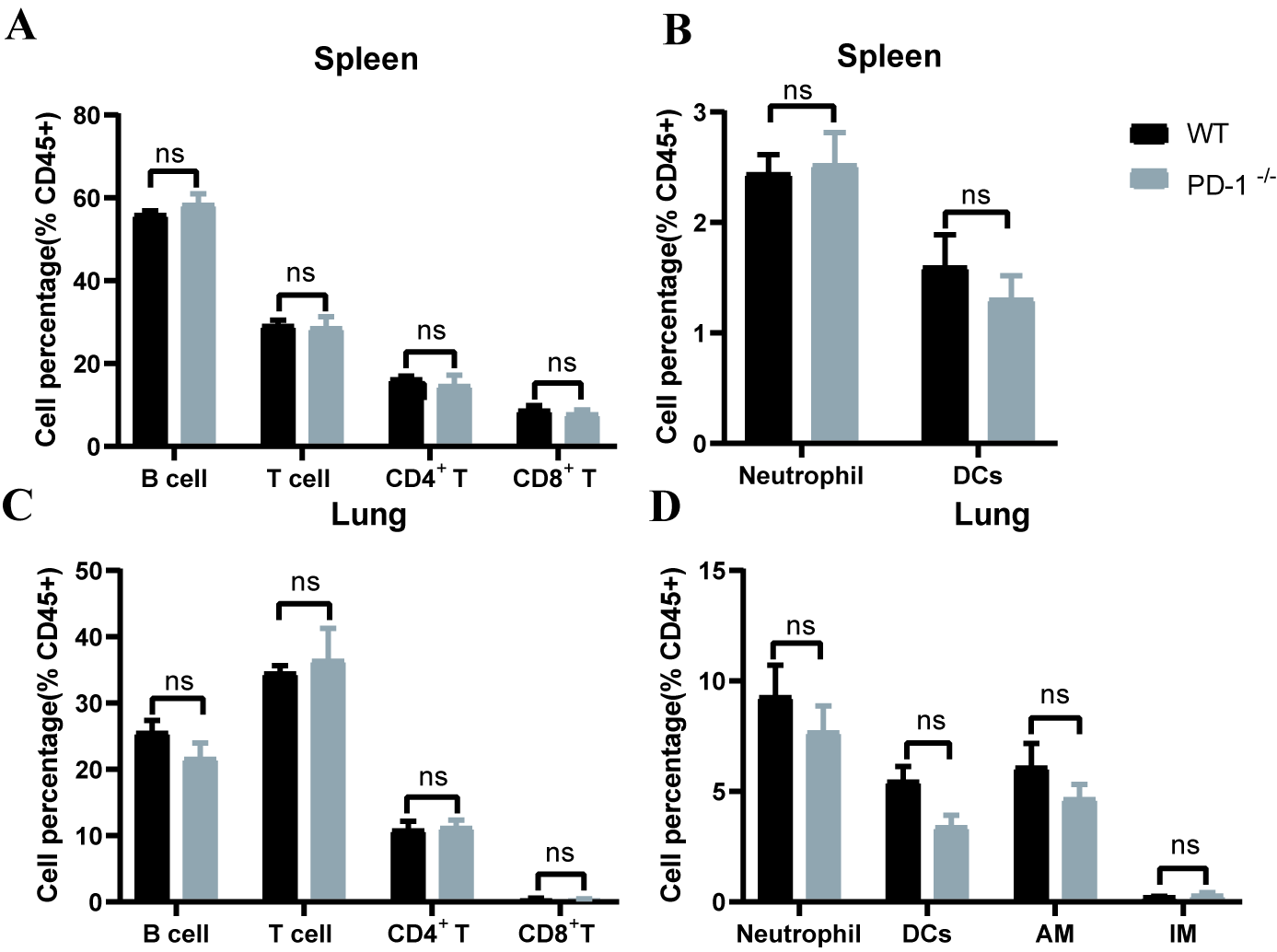

Supplement: Supplementary Figure 3 — Flow cytometric analysis of immune cell composition in the lung and spleen between WT and PD-1−/− mice. (A, B) Immune cell subsets (B cells, T cells, and neutrophils and etc) were quantified by flow cytometry in spleen from WT and PD-1−/− mice. (C, D) Flow cytometric evaluation of immune cell populations (B cells, T cells, and neutrophils and etc) in lung of WT and PD-1−/− mice. (n = 3 per group). [file Image3.tif]

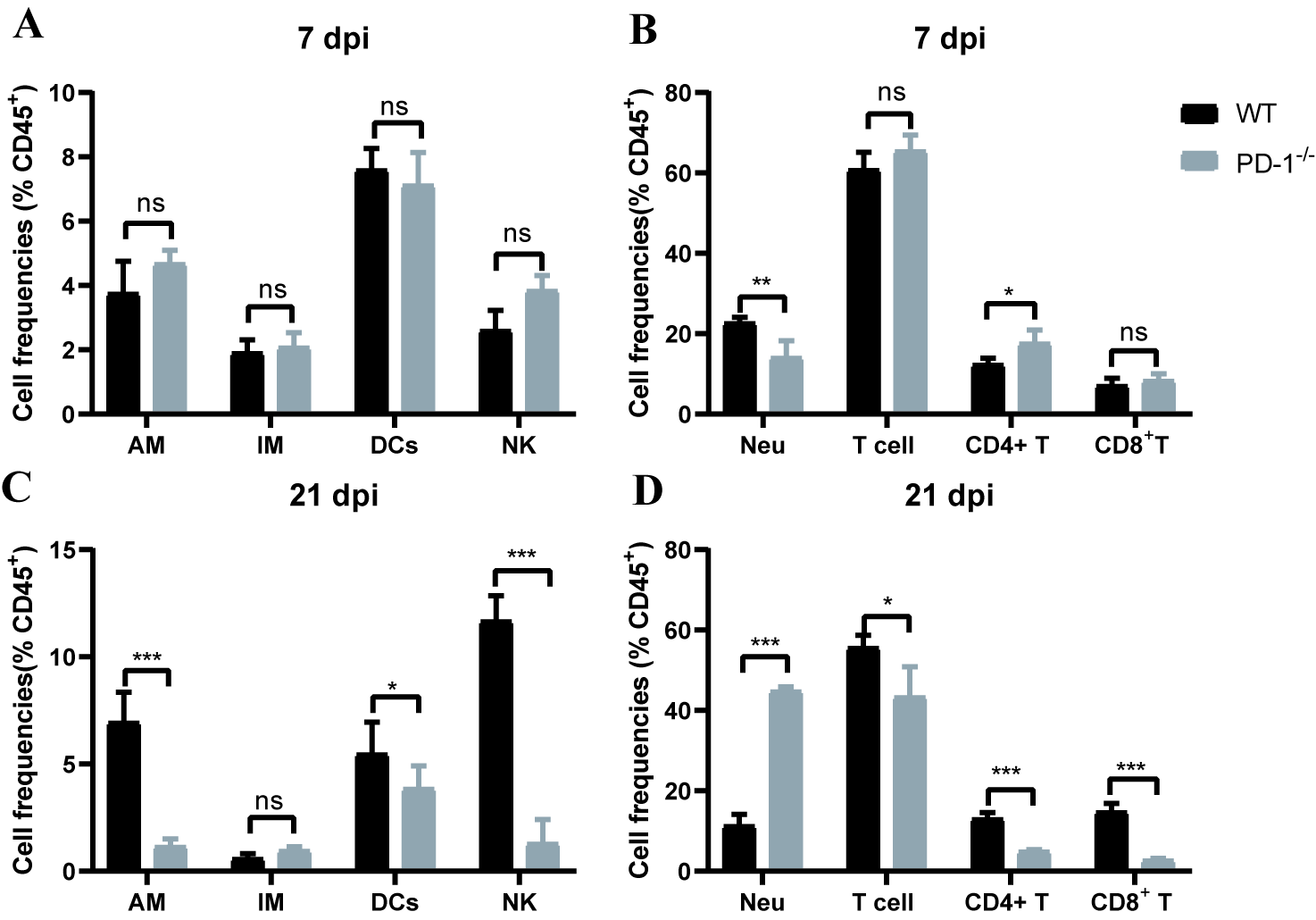

Supplement: Supplementary Figure 4 — Flow cytometric analysis of immune cell populations in lung of WT and PD-1−/− mice at 7 and 21 days post-MAB infection. (A, B) Frequencies of neutrophils, T cells, CD4+ T cells, CD8+ T cells, alveolar macrophages, interstitial macrophages, dendritic cells, and NK cells in lung of WT and PD-1−/− mice at 7 days post-MAB infection. (C, D) Frequencies of neutrophils, T cells, CD4+ T cells, CD8+ T cells, alveolar macrophages, interstitial macrophages, dendritic cells, and NK cells in lung of WT and PD-1−/− mice at 21 days post-MAB infection. (n = 3 per group). [file Image4.tif]

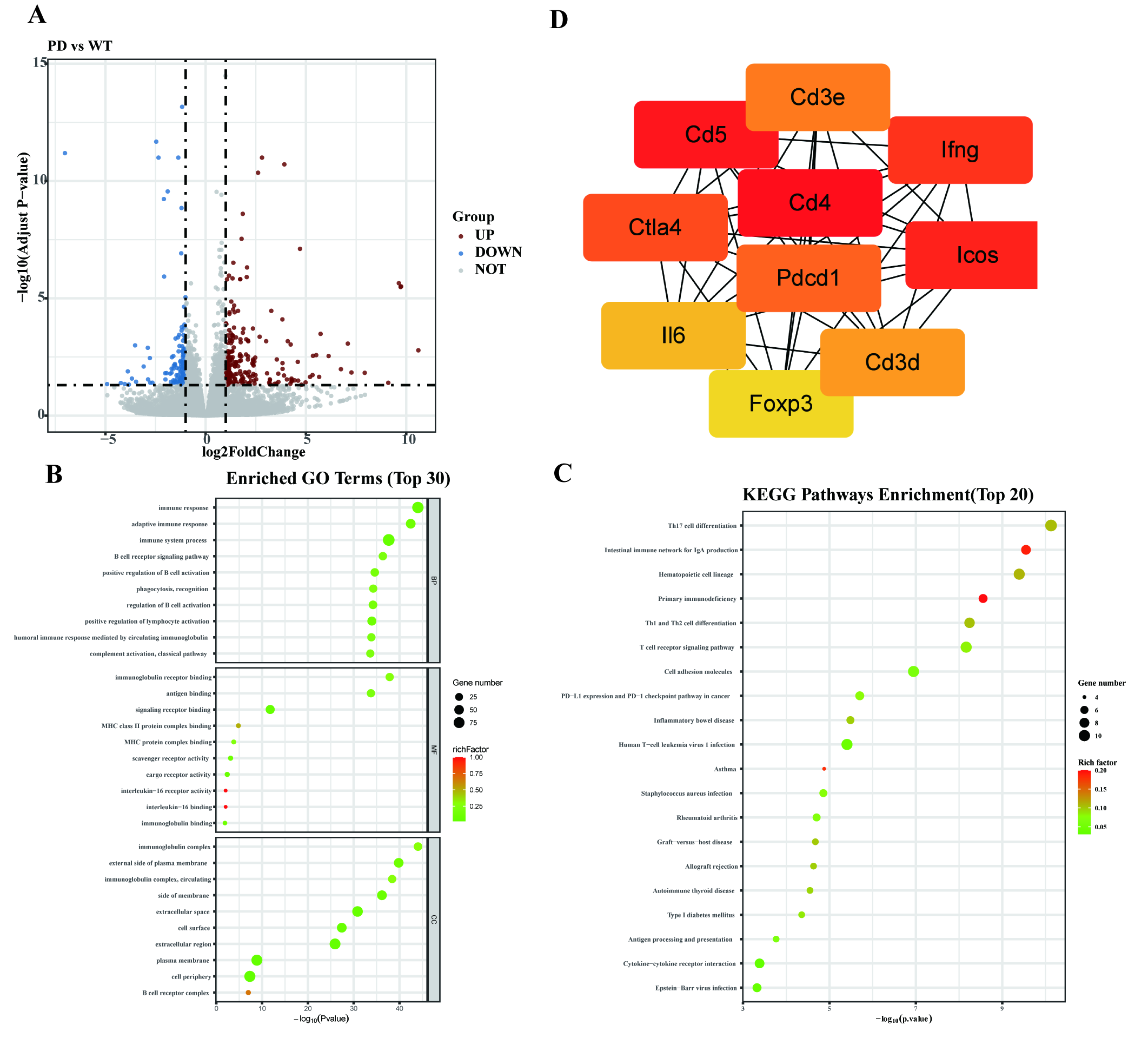

Supplement: Supplementary Figure 5 — RNA-seq analysis of differentially expressed genes in lungs of WT and PD-1−/− mice infected with MAB for 7 days. (A) Volcano plot of differentially expressed genes from RNA-seq data comparing WT and PD-1−/− mice. Upregulated and downregulated genes are highlighted in red and green, respectively. Data are presented as log₂-transformed read counts. (B) GO enrichment analysis of biological processes for upregulated genes. (C) KEGG pathway analysis of upregulated genes in PD-1-deficient mice. (D) Hub genes identified from KEGG pathway analysis of differentially expressed genes in the lungs of WT and PD-1−/− mice after MAB infection. (n = 5 per group). [file Image5.tif]

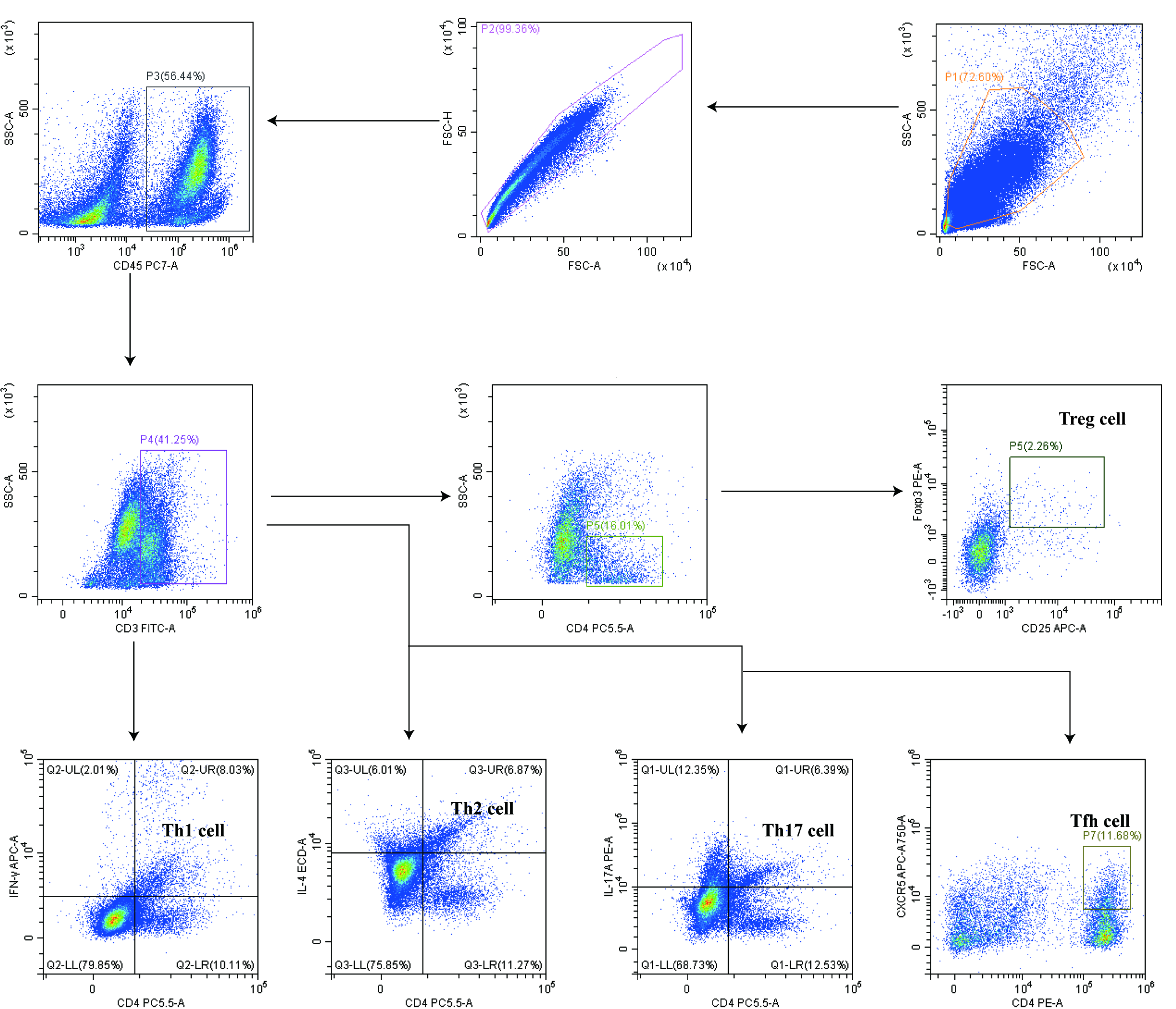

Supplement: Supplementary Figure 6 — Representative flow cytometry gating strategy used to identify Th1 cells, Th2 cells, Th17 cells, Treg cell, and Tfh cells. [file Image6.tif]

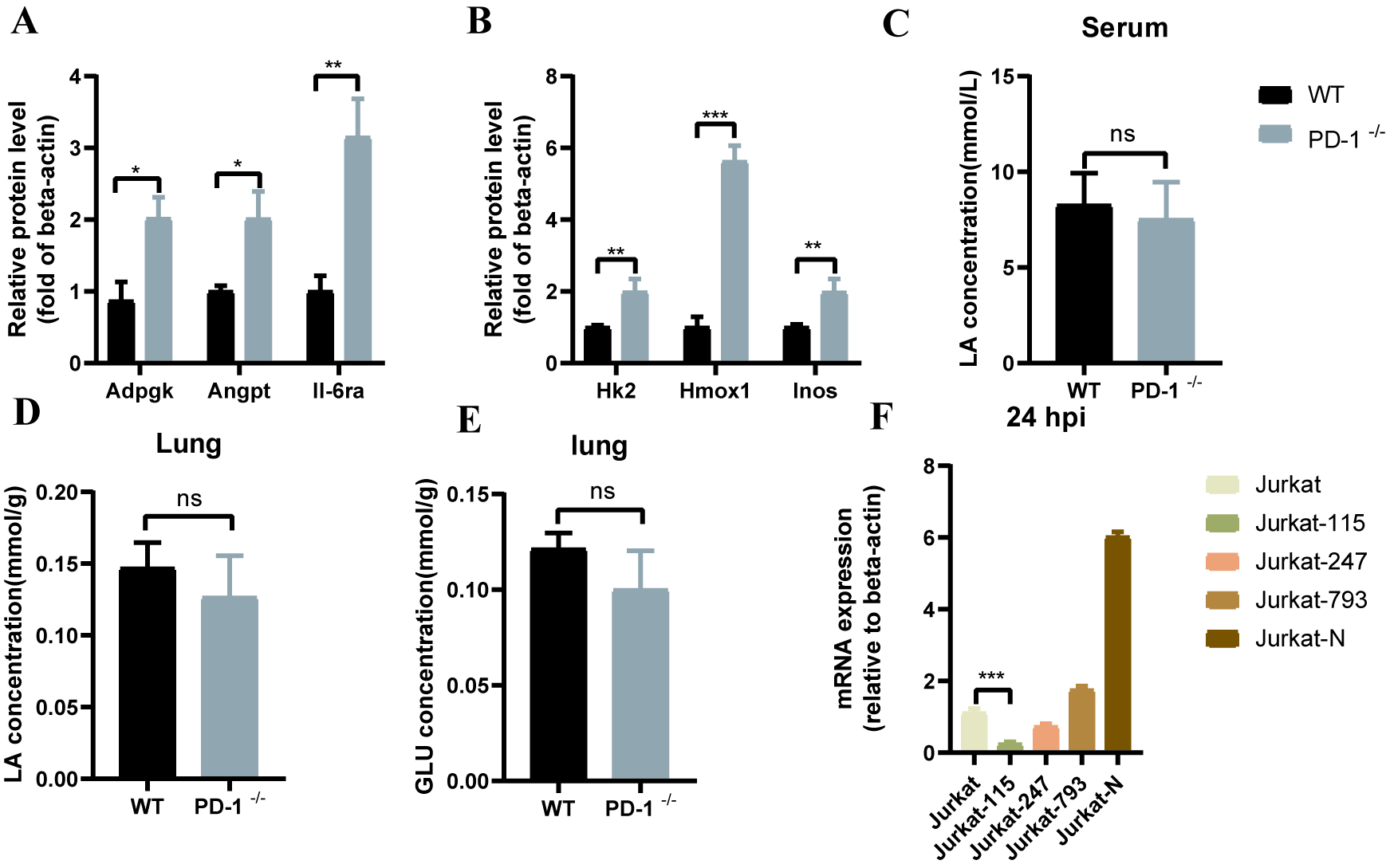

Supplement: Supplementary Figure 7 — Analysis of glycolytic activity and and quantification of HIF-1 signaling pathway proteins in WT and PD-1−/− mice at 21 dpi with MAB. (A,B) Quantitative analysis of HIF-1 signaling pathway proteins expression normalized to β-actin. (C-E) Measurements of serum LA,lung LA and glucose levels in WT and PD-1−/− mice at 21 dpi following MAB infection. (F) Validation of PD-1 knockdown by RT-PCR in Jurkat cells. (n = 3-5 per group). [file Image7.tif]
